# Cheminformatics-based enumeration and analysis of large libraries of macrolide scaffolds

**DOI:** 10.1186/s13321-018-0307-6

**Published:** 2018-11-12

**Authors:** Phyo Phyo Kyaw Zin, Gavin Williams, Denis Fourches

**Affiliations:** 1 Department of Chemistry, North Carolina State University, Raleigh, NC USA; 2 Bioinformatics Research Center, North Carolina State University, Raleigh, NC USA; 3 Comparative Medicine Institute, North Carolina State University, Raleigh, NC USA

## Abstract

We report on the development of a cheminformatics enumeration technology and the analysis of a resulting large dataset of virtual macrolide scaffolds. Although macrolides have been shown to have valuable biological properties, there is no *ready*-*to*-*screen* virtual library of diverse macrolides in the public domain. Conducting molecular modeling (especially virtual screening) of these complex molecules is highly relevant as the organic synthesis of these compounds, when feasible, typically requires many synthetic steps, and thus dramatically slows the discovery of new bioactive macrolides. Herein, we introduce a cheminformatics approach and associated software that allows for designing and generating libraries of virtual macrocycle/macrolide scaffolds with user-defined constitutional and structural constraints (e.g., types and numbers of structural motifs to be included in the macrocycle, ring size, maximum number of compounds generated). To study the chemical diversity of such generated molecules, we enumerated V1M (Virtual 1 million Macrolide scaffolds) library, each containing twelve common structural motifs. For each macrolide scaffold, we calculated several key properties, such as molecular weight, hydrogen bond donors/acceptors, topological polar surface area. In this study, we discuss (1) the initial concept and current features of our PKS (polyketides) Enumerator software, (2) the chemical diversity and distribution of structural motifs in V1M library, and (3) the unique opportunities for future virtual screening of such enumerated ensembles of macrolides. Importantly, V1M is provided in the Supplementary Material of this paper allowing other researchers to conduct any type of molecular modeling and virtual screening studies. Therefore, this technology for enumerating extremely large libraries of macrolide scaffolds could hold a unique potential in the field of computational chemistry and drug discovery for rational designing of new antibiotics and anti-cancer agents.

**Electronic supplementary material:**

The online version of this article (10.1186/s13321-018-0307-6) contains supplementary material, which is available to authorized users.

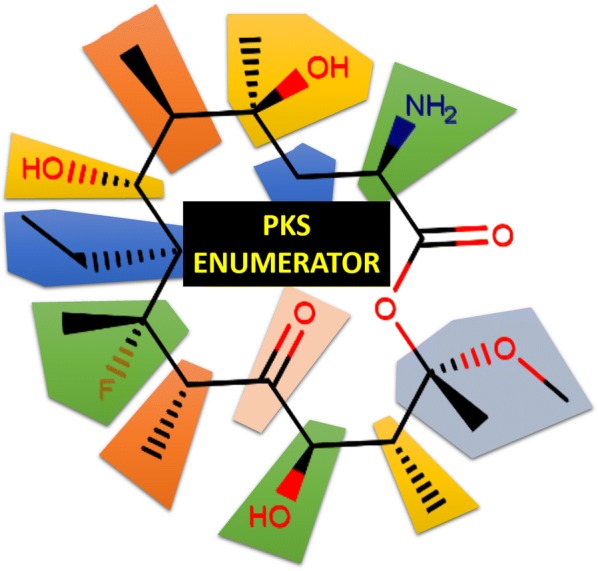

## Introduction

**Fig. 1:**
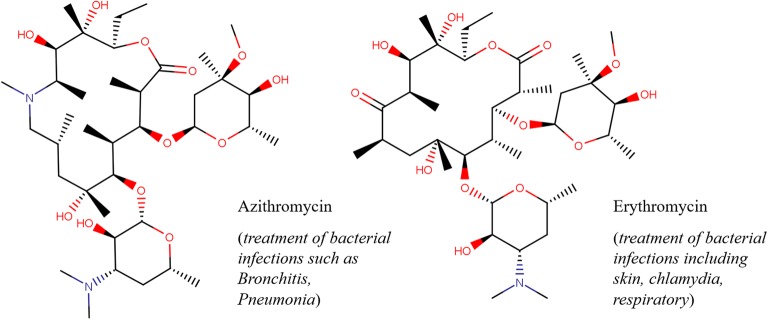
Two examples of well-known bioactive macrolides, Azithromycin and Erythromycin

Macrocycles are ring structures composed of at least twelve atoms in the central cyclic framework [1–3]. Of particular interest are macrolides, i.e., glycosylated macrocyclic lactones belonging to the class of polyketides (PKS) that represent an important family of bioactive molecules [4–6], Macrolides have critical therapeutically-relevant applications [7], such as antibiotics (e.g. Erythromycin, Telithromycin, Clarithromycin, Azithromycin [8]) and anticancer agents (e.g. Dactinomycin, Cyclosporine, Temsirolimus, Sirolimus [8]) (see Fig. 1). Additionally, they have been broadly investigated in modern drug discovery programs as antifungal, antiparasitic, antiproliferative, antituberculosis, and antiviral agents [8].

Macrocycles have been observed to bind difficult protein targets that possess relatively bland surfaces and require large surface contacts [9]. Macrocycles, structurally bigger than small druglike molecules, can better fill and form multiple protein–ligand interactions within these difficult-to-target binding sites [9]. Additionally, as the ring structures of macrocycles can contribute to high structural pre-organization, these features can accommodate for minimal loss of entropic costs [2], and cause macrocycles to usually display binding affinities for diverse biological targets [10, 11]. Importantly, there is heated interest in the investigation of macrolide analogues as tools to chemically probe and manipulate biological systems [11].

One major caveat of exploring macrolide bioactivities in drug discovery is the high difficulty of their organic synthesis. If feasible, synthesis [8, 11, 12] typically requires at least 15 steps and leads to very low yields, consequently impeding the discovery of new bioactive macrolides. Therefore, the prioritization of potent analogues of macrolides is crucial before attempting the experimental synthesis of these challenging organic compounds. To reduce time and financial costs associated with these synthetic efforts, two main avenues are now emerging:

1. *The biosynthesis of macrolides using synthetic biology concepts* Combinatorial biosynthesis is a powerful technology that can produce libraries of unnatural and/or modified structures by genetically manipulating biosynthetic pathways to natural products [4, 13], and has been largely employed to increase chemical diversity of a given molecular scaffold [13]. Major challenges of this technology include limited accessibility of biosynthetic routes and complexities associated with large DNA sequences that contain multiple polyketide synthase genes and other components [11]. One particularly significant consequence of these barriers is the limited structural diversity of polyketides due to the types of structural extender units generated by polyketide producing organisms [11]. To complement these efforts, present-day semi-synthetic strategies rely on finding chemical “handles” to be incorporated into polyketides [11, 14, 15]. The chemical “handles” can then be leveraged via chemoselective ligation chemistries to provide additional chemical diversity. This too has been somewhat restricted in scope and utility [6, 11];
2. *The use of computational chemistry to model, screen, and prioritize the macrolides predicted to have the most promising properties* structure-based virtual screening of chemical libraries towards biological targets has proven capable of identifying novel ligands in a highly cost-and-time effective manner [16–22]. By employing computer-aided tools and heuristics, such as 3D-molecular docking and/or quantitative structure–activity relationship (QSAR) models [21, 22], one could predict the potential activity and synthetic feasibility of compounds in order to prioritize analogues with desirable structural and molecular properties. Cheminformatics approaches could thus be highly relevant to further the use of novel macrolides in pharmaceutical development. In fact, they could facilitate and speed up the identification of promising compounds to be biosynthesized in priority.

Molecular docking and QSAR methodologies have indeed proven useful and reliable enough to design and/or identify highly potent molecules with desired biochemical properties and binding bioprofiles [16–18, 20–25]. Thus, we posit that high-throughput virtual screening could help researchers discover valuable molecules from a library of enumerated analogues. Consequently, the overall cost associated with the design and synthesis of those bioactive macrolides could be dramatically reduced. Obviously, this is especially true if most of those enumerated macrolides can be biosynthesized by engineering polyketide synthase modules. However, to achieve this ambitious goal, there is an urgent need for the creation of large and diverse virtual chemical libraries of macrolides that could be virtually-screened against a given biological target of interest (e.g., bacterial ribosome) or a series of already known bioactive macrolides treated as active probes. As far as we know, there is no large library of macrolides ready for virtual screening in the public domain. Therefore, developing new cheminformatics tools to generate large sets of virtual macrolide scaffolds represents a valuable resource for computational modeling and virtual screening of novel bioactive compounds.

**Fig. 2:**
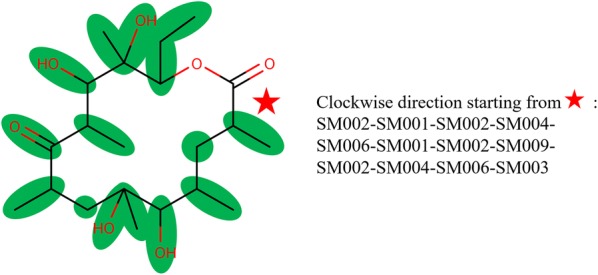
An example macrolide scaffold with twelve structural motif (SM) units as building blocks. The sequence of SMs indicates their building order in the structure. The associated chemical names and structures of SMs can be found in Fig. 4

**Fig. 3:**
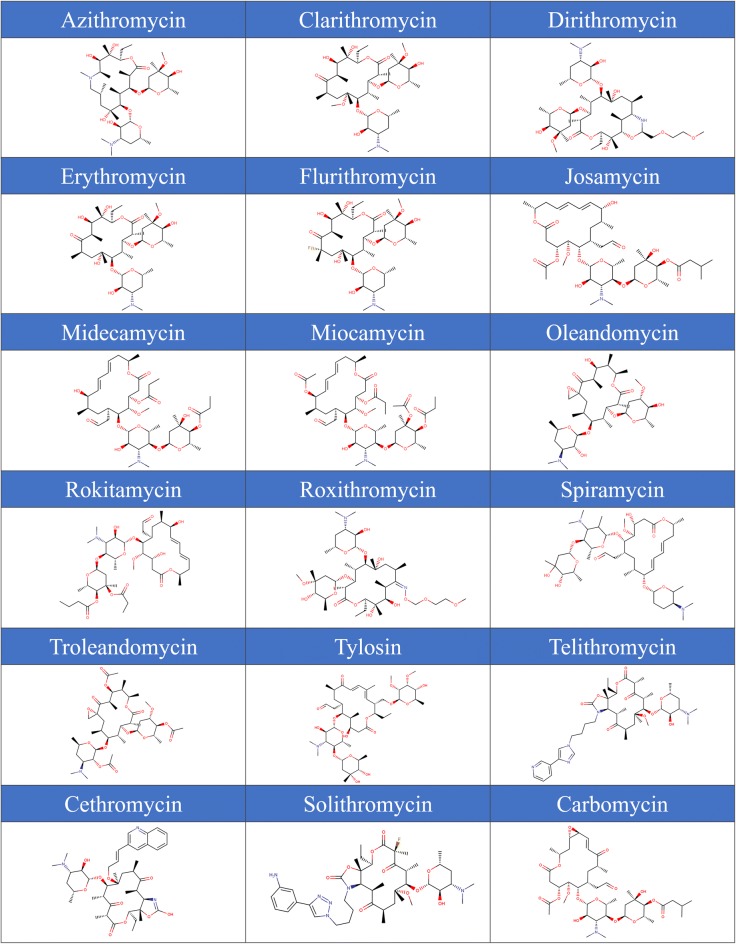
List of eighteen well-known bioactive macrolide drugs. These structures were later simplified by removing sugar groups and replacing ester and amino chains protruding from the core cyclic structures with alcohol and amine respectively (see Supplementary Figure S2)

The main goal of this study was to develop and test a computational approach to enumerate extremely large libraries of macrolide scaffolds involving “common” and “rare” structural motifs. Structural motifs (SMs) are envisioned as the constitutional building blocks (highlighted green in Fig. 2) at the foundation of a fully assembled macrolide scaffold. In our approach, SMs are added one by one and permuted to create and enumerate new macrocycles with all possible arrangements of SMs. SMs currently employed in our software were directly and solely derived from eighteen known, experimentally-confirmed bioactive macrolide scaffolds compiled from different studies (Fig. 3) [26–34]. The approach currently has nine “common” structural motifs and seven “rare” structural motifs. That restriction dramatically enhances the likelihood of having these macrolide scaffolds to be successfully biosynthesized if found to have promising properties. Herein, we conceived and implemented the PKS Enumerator program capable of generating large, highly customized virtual chemical libraries of macrocycle/macrolide scaffolds with controlled structural diversity. It can serve as a *unique*-*in*-*its*-*genre* tool to explore the chemical space of virtual macrocycles, investigate their chemical/physical features, and create well-designed libraries of macrocycles ready for virtual screening. Obviously, the vast majority of those macrocyles will never be (bio)synthesized due to the time and cost such an endeavor would represent. However, having the possibility to study the structural properties of those large series of macrolide scaffolds is of high interest for modelers and computational chemists. Besides, our PKS Enumerator technology can be coupled with other cheminformatics software such as BoBer which replaces some of the isosteric fragements to improve overall potency, reduce toxicity, and change bioavaility [35]. Moreover, the enumerated libraries of virtual molecules can have other uses when it comes to mapping and studying the chemical space with computational techniques.

**Fig. 4:**
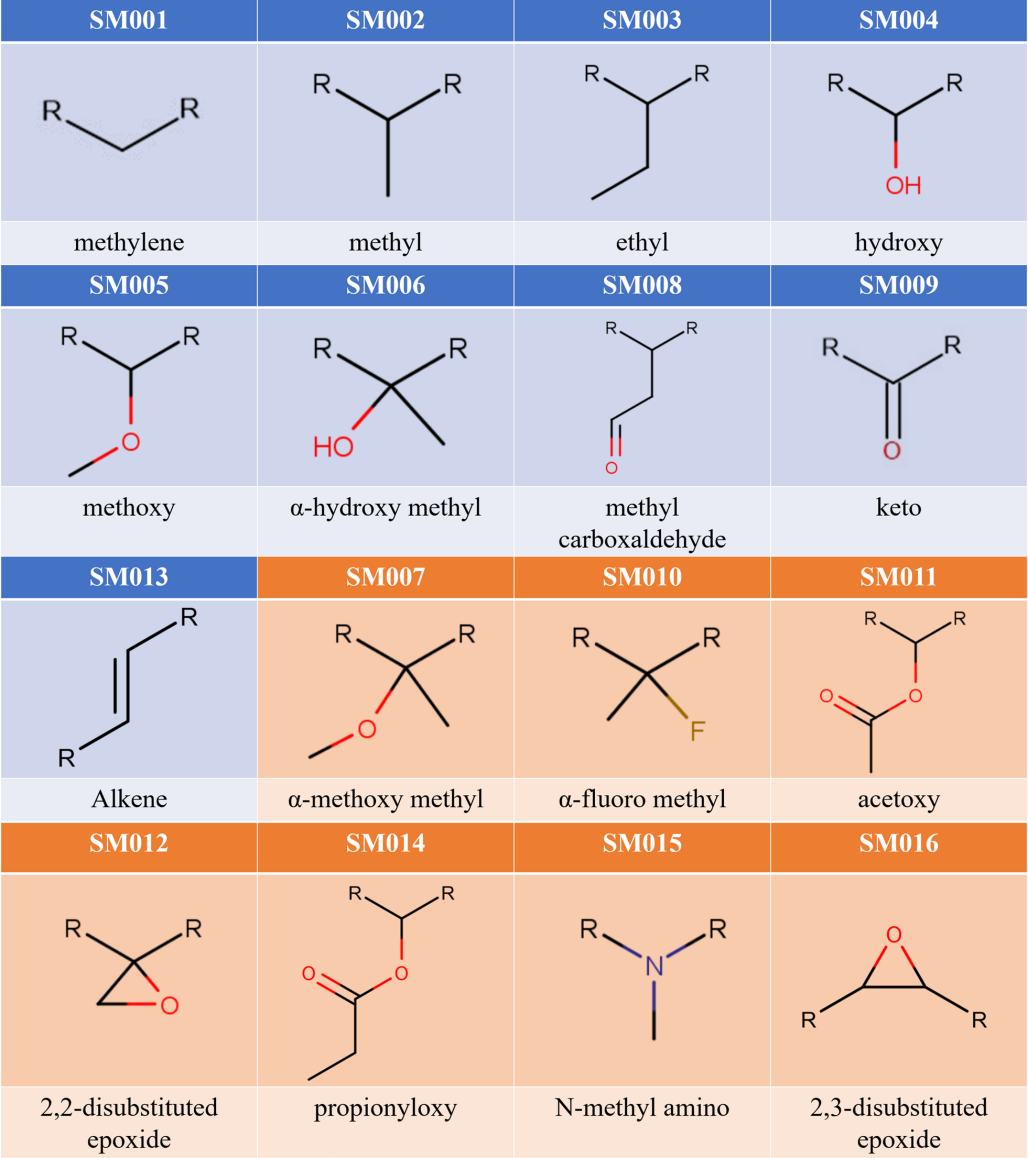
Sixteen structural motifs currently employed in PKS Enumerator. SMs were retrieved from eighteen known bioactive macrolide scaffolds (Fig. 3): nine “common” structural motifs (CSMs) are in blue cells, and seven “rare” structural motifs (RSMs) are in orange cells

As the main case study, we report on the enumeration and structural characterization of the V1M library, a sample library generated via PKS Enumerator containing 1 million diverse macrolide scaffolds built using nine common SM types (blue cells in Fig. 4). It was directly inspired by the core structures of eighteen known bioactive macrolides. Again, our overarching goal is to generate virtual macrolide scaffolds that share high structural similarity with well-known bioactive macrolides. We then analyzed the distributions of SMs along with several molecular descriptors (molecular weight, hydrophobicity, topological polar surface area, hydrogen bond donors/acceptors, rotatable bonds, hetero atoms, heavy atoms) calculated for each enumerated macrolide scaffold. We also conducted fingerprint analysis of V1M to determine the level of chemical similarity towards the eighteen bioactive macrolides.

Overall, V1M was generated as a proof-of-concept study (1) to help illustrate the features of PKS Enumerator, (2) to demonstrate how chemical and structural diversity can be adeptly controlled with the user-provided parameters, and (3) to encourage computational (and potentially experimental synthetic) scientists to custom design virtual chemical libraries of macrolide scaffolds suited for their project needs. V1M is freely available in the Supplementary Material of this manuscript (Additional file 1). Moreover, the PKS Enumerator is also freely available for download (http://www.fourches-laboratory.com/software). We believe this new virtual library of publicly available macrolide scaffolds will enable and inspire other molecular modeling studies.

## Methods

### Overview of the enumeration system

PKS Enumerator is a novel cheminformatics software that enumerates and generates virtual chemical libraries of macrocycles. The innovation of our enumeration approach relies on its ability to create extremely large and diverse chemical libraries by manipulating and constraining key structural parameters of the enumerated compounds, such as the type, number, and redundancy of structural motifs in each compound or the overall diversity of the library. The software itself has been developed in Python 3.5 and can be accessed via a graphical user interface. The software is freely available for download (http://www.fourches-laboratory.com/software) including its most recent GUI (https://github.com/zinph/pks-enumerator).

**Fig. 5:**
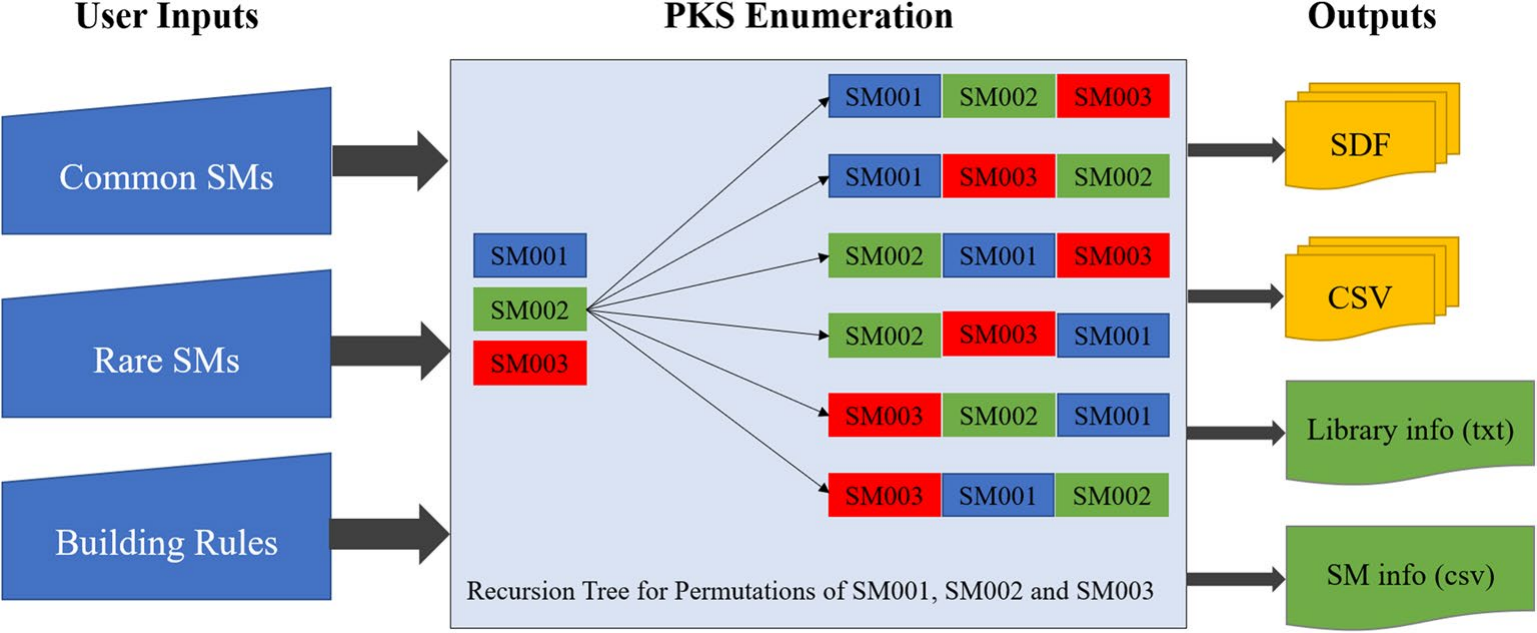
Simplified workflow diagram of PKS Enumerator system

An example macrocycle, with ring size twelve, is shown in Fig. 2. One should note that “ring size” here is defined as the number of SM units included in the ring structure. As illustrated by its simplified workflow diagram (Fig. 5), PKS Enumerator allows for the integration of two types of structural motifs: *common* and *rare*. The core workflow of our PKS Enumerator is provided and explained in depth in the “Implementation details” section. The basic building blocks, i.e., structural motifs, in our method are functional groups such as alkenes, epoxides, esters, carboxylic acids, etc., with identified joining points (represented with “R”s in Fig. 4) at which other SMs will be connected. These blocks are utilized to form the core ring structures of macrolide scaffolds and are major contributors to the structural diversity of the libraries generated.

In this approach, SMs were directly and solely derived from eighteen known, experimentally-confirmed bioactive macrolide scaffolds compiled from different studies (Fig. 3) [26–34]. “Common” structural motifs (CSMs), in this context, were found in at least five out of eighteen known bioactive macrolide drugs, while “rare” structural motifs (RSMs) were found in less than five (Additional file 2: Figure S1). This is very important as our primary goal was to generate macrolide scaffolds that primarily include biosynthetically-amenable building blocks found in known bioactives. We believe this will increase the feasibility of such virtually generated compounds through combinatorial biosynthetic approach [11, 14, 15]. One should also note that both CSM and RSM categories could be expanded, i.e., our program is not limited to nine CSM types and seven RSM types reported in this study. Other SM types can be hardcoded into the software per request. On that premise, RSM category may eventually include SMs that are difficult to synthesize and/or insert into a macrolide scaffold, and we believe such an option is useful for future molecular design and exploratory studies.

The list of nine CSM types and seven RSM types employed for enumeration are provided in Fig. 4. Once a subset of these basic building blocks has been selected by the user, PKS Enumerator automatically imports them into the program. Using SMs as building blocks, the software permutes and creates macrocycles according to the filters and constraints set by the user (see Table 1). All generated structures are exported as SDF (with two-dimensional structures) and CSV files, with six molecular properties calculated for each compound: molecular weight—MW, hydrophobicity—SlogP, topological polar surface area—TPSA, hydrogen bond acceptors—HBA, hydrogen bond donors—HBD, and rotatable bonds—NRB. These properties were selected because they are often used to indicate drug likeness, cell permeability, and oral availability of molecules.

**Table 1:** Constraints used in PKS Enumerator. User inputs for generating V1M library were also provided

Building rules	V1M inputs	Parameter IDs
Range of CSMs in each macrocycle		
Minimum	12	1
Maximum	16	2
Range of RSMs in each macrocycle		
Minimum	0	3
Maximum	0	4
Range of total structural motifs in each macrocycle		
Minimum	12	5
Maximum	12	6
Prioritize “common” or “rare” category	CM	7
Number of permutations to skip	100,000	8
Library size	1,000,000	9
Addition of an ester	Yes	10
Repetition of each CSM and RSM	(varies) Fig. 5	11

The key user-defined constraints and filters in our program regarding the structural characteristics of macrocycles are recapitulated in more details in the next section. Briefly, users can choose to have an additional ester in the macrocycles, specify the types of CSMs, RSMs to build the core macrocyclic structures, and fix the number of SMs for each macrocycle. Additionally, users can control the overall ring sizes and the library size (e.g., 100 million unique compounds). The diversity of the library can also be controlled by assigning the number of permutations to be skipped after each macrocycle is written to the output file.

### User controls and enumeration of V1M library

**Fig. 6:**
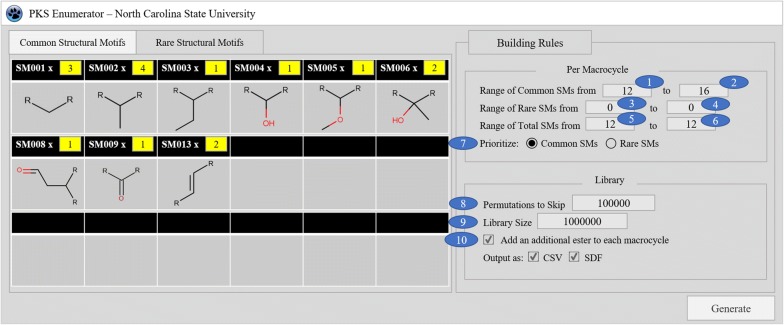
Graphic user interface of PKS Enumerator software. The numbers in the blue circles correspond to the parameters explained in the “Methods” section. Yellow cells in the structural motif cells indicate the maximum repetitions allowed for the corresponding SMs

We recapitulate in Table 1 the parameters used to set up the requirements for our program execution. A preliminary graphic user interface of PKS Enumerator software, along with nine currently employed CSMs and building rules, is provided in Fig. 6. The example inputs are taken from the parameters we used to enumerate V1M, reported and analyzed in the “Results” section.

First and second parameters control the allowed range of CSM units to be included in each macrocycle. Since numerical values of 12 and 16 have been provided for minimum and maximum number of CSMs, macrocycles can contain twelve to sixteen CSM units per ring if the total ring size set in fifth and sixth parameters permits. Similarly, third and fourth parameters determine the minimum and maximum number of rare structural motif (RSM) units allowed per macrocycle. For V1M, these inputs have been set to 0; therefore, macrocycles will not contain any RSMs. The fifth and sixth parameters, respectively, determine the minimum and maximum numbers of building blocks or SM units to be included in each macrocycle; in other words, the ring size. The program will generate all ring sizes of macrocycles starting from the minimum value, incrementing up to the designated maximum number. Since both minimum and maximum inputs are twelve in the example provided in Table 1, only macrocycles with twelve SM units will be generated. The inputs for the above parameters must be coherent for the program to work as desired. For example, if the minimum number of total SMs (5th parameter) is higher than the combination of specified maximum CSMs and RSMs (2nd and 4th parameters), the program will not generate any compound and return an error message. This is because the sum of maximally allowed CSMs and RSMs is lower than the required minimum ring size. Regardless, ring size (5th and 6th parameter) takes precedent over the range of CSMs and RSMs allowed per macrocycle (1st, 2nd, 3rd and 4th parameters).

The seventh parameter determines whether CSM or RSM categories will be prioritized. If users choose to prioritize CSM, the permutation process will start with maximally allowed number of CSMs and decrement until the minimally allowed CSMs per macrocycle. In other words, the library will exhaust all possible arrangements of CSMs before including RSMs in macrocycles. Hence, a library may contain macrocycles with only CSMs upon one or more of the following conditions which can potentially limit RSMs from partaking in the permutation process: (1) large subsets of CSMs are imported, (2) small skipping parameter is provided, or (3) library size is set too small. The reverse can happen if RSMs are prioritized over CSMs.

The eighth parameter controls the number of macrocycles to be overlooked after each has been written to the output file. Once the program starts, it enumerates the first permutation of the building blocks to generate one macrocycle and writes the resulting structure to the output file if the specified structural requirements are met. Then, the program skips a specified number of permutations per user’s request, and repeats the process of enumerating, checking and writing the compounds. This process continues until the desired library size is achieved or no more permutations are left to continue. Since macrocycles are built via block permutations in a standardized and linear order (see recursion tree in Fig. 5), larger inputs for the skipping parameter delivers higher diversity. This option is particularly helpful when one desires to generate a very diverse yet representative library covering an extremely large portion of the chemical space potentially buildable using all the selected SMs.

The ninth parameter determines the total number of macrocycles to be generated at the end of program execution. In other words, this parameter controls the overall size of the virtual chemical library. It is necessary for efficiency of the software as well as the amount of storage. In V1M, the total number of macrocycles is limited to 1 million. The tenth parameter indicates whether each macrocycle must contain an additional ester or not; the former produces macrolide scaffolds, and the later macrocycle scaffolds. The eleventh parameter (yellow cells in Fig. 6) controls how often each SM type can be repeated per macrocycle; in other words, users can specify the maximal occurrences of each SM type. This parameter is useful because repetition of certain CSM types, such as SM001 and SM002, was observed in eighteen bioactive macrolides (Additional file 2: Figure S1A, S1B).

Since hundreds of millions of macrocycles can be exported, output files are separated and organized based on their ring size (total structural motif units). In this example, since the ring size was twelve, the output files were named ‘RS_12.csv’ and ‘RS_12.sdf’ where ‘RS’ stands for ring size. Additionally, the program outputs two other files containing important information about the library: library_info.txt and SM_info.csv. The former has a compilation of all the input parameters set for the library, along with the selected CSMs, RSMs and time elapsed for the enumeration process. The later reports data on CSM and RSM type distribution along with their number of repeats per macrocycle (outputs in Fig. 5).

## Implementation details

**Fig. 7:**
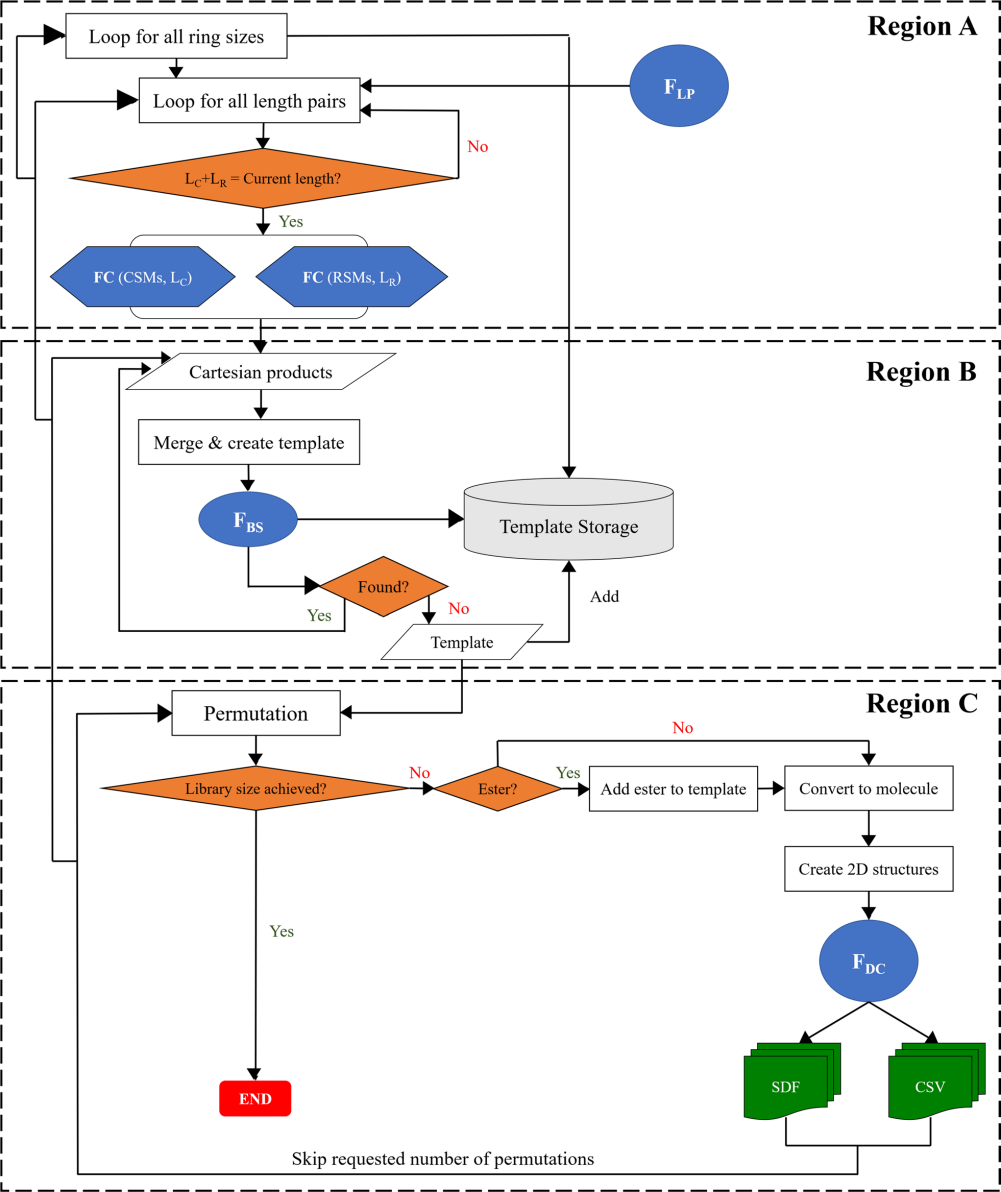
General workflow of the core PKS Enumeration process. Regions A, B and C are explained in the Implementation Details of the “Methods” section

The core algorithm of PKS enumeration process is presented in Fig. 7. The cheminformatics backbone of this python script relies on the RDKit library [36] and PKS_Enumeration object. The later employs four helper methods (F_BS_, F_DC_, F_LP_, F_C_) provided in Table 2 along with comprehensive descriptions to help aid in understanding the workflow of the algorithm. Three major inputs required for the program are CSM types, RSM types, and a list which compiles the building rules, i.e., all eleven parameters from in “Methods” section. CSMs and RSMs are shuffled after being imported into the program to prevent the repeatable SMs from clustering together.

**Table 2:** Helper functions from PKS_Enumerator class. The descriptions for each function were provided to help understand the workflow of PKS Enumerator provided in Fig. 7

Methods	Description
Binary search function (F_BS_)	Searches target item in the given list. Implemented to ensure no duplicate macrocycles are generated
Descriptor computing function (F_DC_)	Computes six molecular descriptors for the input compound via RDKit library: Molecular Weight—MW, Hydrophobicity—SlogP, Hydrogen Bond Acceptors—HBA, Hydrogen Bond Donors—HBD, Topological Polar Surface Area—TPSA, and Rotatable Bonds—NRB
Length pair function (**F**_**LP**_)	Generates all possible common and rare SM length arrangements based on total number of SMs allowed in the program. This method is reliant on parameters 1, 2, 3, 4, 5, 6 and 7 in the building rules. The length pairs returned are sorted based on ascending number of either common or rare structural motifs (7th parameter). Default prioritizes CSMs, i.e. length pairs are sorted based on ascending lengths of rare SMs, thereby prioritizing CSMs in the macrocycles. In each length pair, the number of CSMs are noted as L_C_ and RSMs as L_R_. Their sum provides the total number of SMs per macrocycle
Combination function (**F**_**C**_)	Generates all different combinations of SMs per input length

First (Fig. 7, Region A), the program generates all possible ring sizes of macrocycles based on 5th and 6th parameters. For each ring size, it generates all possible allowed length pairs of CSMs and RSMs per macrocycle based on 1st, 2nd, 3rd and 4th parameters using length pair function (F_LP_). For each ring size, an empty template storage is created to hold templates containing CSMs and RSMs in string representations (SMILES). These stored templates are later parsed with a binary search function (F_BS_) to ensure that no duplicate macrocycles are produced. In other words, duplicate check is performed on canonical SMILES by searching the template storage of associated ring size. Here, the orders of CSMs and RSMs in those templates do not matter, only that the same SMs are present in those templates. Then, length pairs of CSM and RSM, L_C_ & L_R_ (L_C_ = allowed CSM units and L_R_ = allowed RSM units), are iterated for each ring size, and checked to ensure that the pair adds up to the current ring size. If the length pair does not add up, it simply continues to the next length pair. This step confirms macrocycles with only the user-specified numbers of CSMs and RSMs per ring size to be generated in the library. For the desired length pairs, all possible combinations for selected CSMs and RSMs per their respective allowed lengths are generated using the combination function (F_C_). This will generate two separate categories: CSMs combinations per L_C_ and RSMs combinations per L_R_.

In the next step (Fig. 7, Region B), cartesian products (CP) of CSM and RSM categories are generated. Here, CPs are essentially templates holding varying mixtures of CSMs and RSMs. At this stage, the positions of SMs in the template are of little significance because the template is then sorted and binary search function (F_BS_) is applied to perform duplicate check in the associated template storage as mentioned above. If the template with the same SMs is not found, it is added to the template storage, and then passed into the permutation process, during which the actual permutation of all SM blocks is conducted to create macrocycles with different arrangements of SMs (see recursion tree in Fig. 5). If the template exists already, it will simply move to the next CP, repeat the process of creating a template, and checking its existence in the template storage.

During the permutation process (Fig. 7, Region C), the program will first check whether the library size (input from 9th parameter) has been achieved. If so, the program will end. If not, the template (canonical SMILES) will be standardized based on molvs module [37], and an ester may or may not be added per user’s request (10th parameter). Molvs standardization is performed by removing hydrogens, sanitizing mols, disconnecting metals, normalizing, reionizing and assigning stereochemistry [37]. Next, the formatted template, containing SMs, is converted into a compatible RDKit mol format, for which 3D-coordinates and conformations may or may not be generated using ETKDG method from RDKit library [36] upon user’s request. Descriptor calculation function (F_DC_) is then performed on the molecule (see Table 2), and the macrocyclic compound will be written to SDF (with or withour generation of 3D coordinates per user’s request) and CSV files. Specified number of permutations (8th parameter) will then be skipped after the molecule has been written to the output file. The permutation process will continue until all possible permutations for this template has been completed, after which, it will loop to the next cartesian product which will provide another template to permute.

## Results

For this *proof*-*of*-*concept* study aiming at generating the V1M library, we constrained the program so that each enumerated macrolide scaffold had a total of twelve SM units (i.e., to create 14-member ring macrolide scaffolds such as Erythromycin). Each SM unit was selected from the nine unique CSM types (see inputs for V1M in Table 1 and Fig. 6). SM001, SM002, SM006, SM013 were allowed 3, 4, 2 and 2 times per macrolide scaffold respectively, and the remaining CSM types once. These values were chosen by an approximate weighting based on the frequency of CSM types found in each of the eighteen bioactive macrolide (BM) scaffolds (see Additional file 2: Table S1). In other words, we allowed frequently occurring structural motifs such as SM001 and SM002 to be repeated more often than the others. We had the program skip 100 k permutations after each output structure. An additional ester was added, and V1M was set up to contain exactly 1 million macrolide scaffolds (Table 1).

The number of all possible macrolide scaffolds according to the input parameters used for V1M was approximately 872 billion compounds, since each macrolide scaffold used twelve SMs taken from a total of sixteen available CSMs in the selection pool (i.e., 3 repeats of SM001, 4 repeats of SM002, 2 repeats of SM006, 2 repeats SM013, 1 repeat of SM003, SM004, SM005, SM008, SM009). Herein, we generated a total of 1 million compounds by skipping 100 k possible compounds after each selected macrolide scaffold. Considering that all the examined compounds met the required structural features and were chosen to be in V1M, we only covered the first 100 billion compounds, i.e., a small fraction (11.47%) of the entire possible macrolide scaffold population based on the user-defined constraints. It took a standard desktop PC (Intel^®^ Core™ i7 CPU, 8 GB RAM) approximately seven hours to generate V1M. Then we analyzed it in terms of:

- *Structural diversity* We reported on the distribution, composition of CSM types, their occurrence(s) per macrolide scaffold, along with the distribution of heteroatoms and heavyatoms observed in V1M. We computed the same properties for simplified structures of eighteen well-known BM drugs (Additional file 2: Figure S2) and compared their results to those of V1M.
- *Chemical diversity* We studied the distributions of molecular descriptors (molecular weight—MW, hydrophobicity—SlogP, topological polar surface area—TPSA, hydrogen bond acceptor—HBA, hydrogen bond donor—HBD, rotatable bond—NRB) which are commonly used to assess drug likeness, bioavailability, and oral absorption [38–43]. Additionally, we computed the same descriptors for eighteen BM scaffolds, and conducted a comparative analysis to further emphasize the chemical diversity of V1M.
- *Correlation Analysis* we conducted a pairwise correlation analysis among all computed descriptors (MW, SlogP, TPSA, HBA, HBD, NRB, heteroatoms, heavyatoms).
- *Structural similarity with eighteen BM scaffolds* we assessed the chemical similarity of our 1M macrolide scaffolds from V1M with respect to the eighteen BM scaffolds by conducting fingerprint analysis (MACCS).

Regarding the comparative analyses with the eighteen BMs, substituted ester and amino functional groups protruding from the ring cyclic frameworks were replaced with alcohol and amine groups respectively, and sugar blocks were removed. This preprocessing step allowed us to directly compare the molecular properties of the scaffolds of the well-known BMs to those generated in V1M. Simplified structures of the eighteen BMs are provided in Fig. 2. For example, the structure of Erythromycin was modified as shown in Additional file 2: Figure S3.

### Structural diversity of V1M

**Fig. 8:**
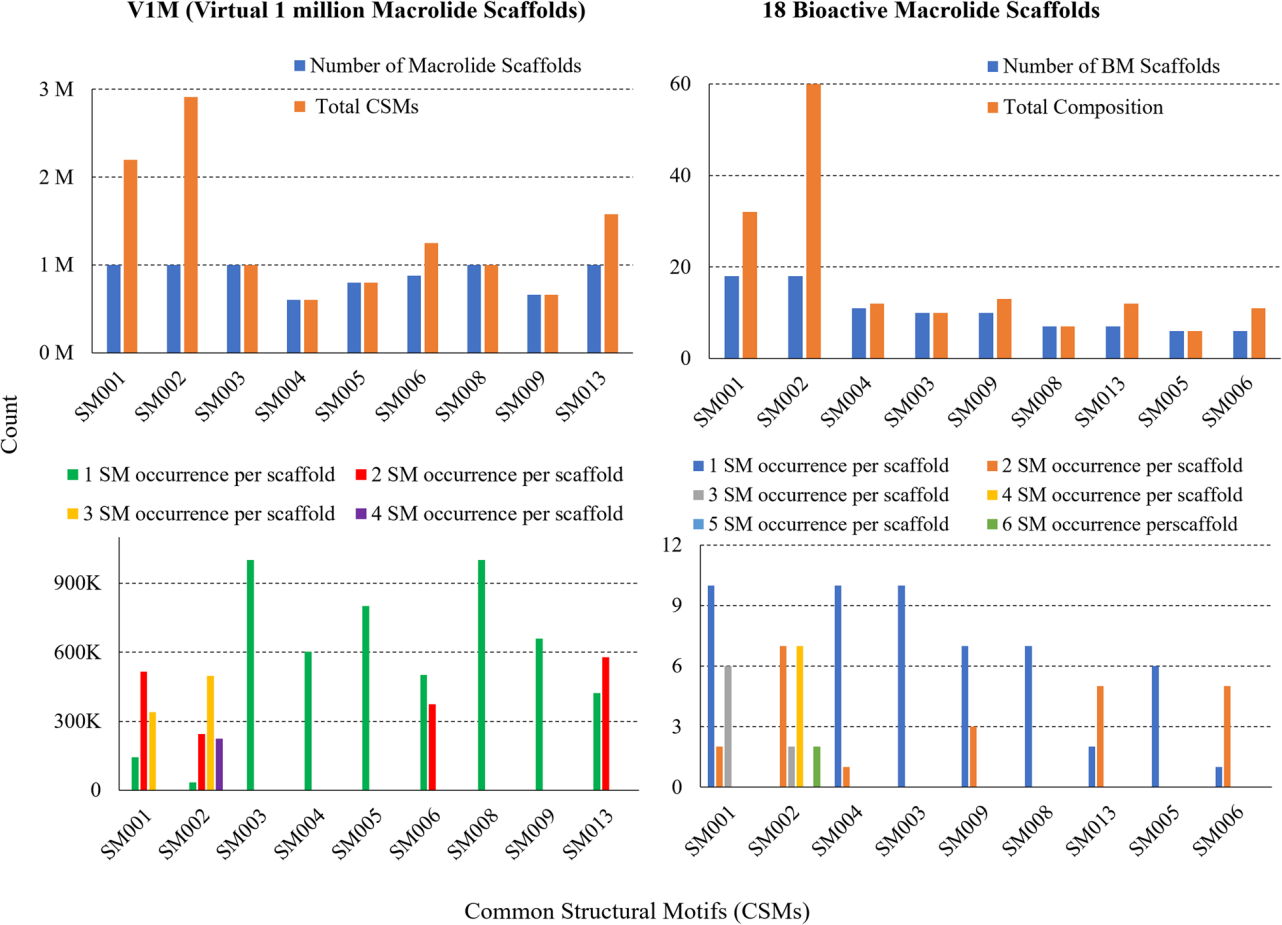
Distribution of structural motifs in **a** V1M (Virtual 1 million Macrolide scaffolds), and **b** 18 BM (Bioactive Macrolides) scaffolds from Figure S2. Blue represents the number of macrolide scaffolds with specified SM types. Orange represents the total composition of SM types in the entire library, accounting for their repeats per macrocycle scaffold. Distribution of SM occurrences per macrolide scaffold in **c** V1M, and **d** 18 BMs

We analyzed the structural diversity of V1M by studying the distribution of CSM types, each type’s composition, the occurrence of each CSM type per macrolide scaffold, counts of heteroatoms and heavyatoms. The distribution of CSM types and their composition in V1M were presented in Fig. 8a. The blue bars represent the number of macrolide scaffolds where respective CSM types are observed, and the orange bars represents the total composition of each CSM type in the entire library. All nine CSM types, in general, were highly represented (blue, Fig. 8a); SM001, SM002, SM003, SM008 and SM013 were observed in all 1M macrolide scaffolds, and SM006, SM005, SM009, SM004 were observed in 875 k, 800 k, 660 k, 602 k macrolide scaffolds respectively. V1M employed all nine unique CSM types among a selection pool of sixteen CSMs; therefore, the overall diversity of SMs in V1M is significantly high. Among the eighteen BM scaffolds, we observed a somewhat similar distribution of CSM types (blue, Fig. 8b). All nine CSM types were present in eighteen BM scaffolds. SM001 and SM002, like in V1M, were involved in all eighteen BM scaffolds; SM003, SM005, SM006, SM008 and SM013 were found in ten, six, six, seven and seven bioactive macrolide scaffolds. Interestingly, SM002 was observed twice more frequently than SM001 in these eighteen BM scaffolds. We could probably posit that the methyl structural motif (SM002) helps maintain/impose critical conformational constraints for the macrolides (compared to the SM001). The direct comparison of CSM type distribution (in percentages) among the scaffolds of the eighteen BMs and V1M was shown in Additional file 2: Figure S4A.

We then studied the total composition of CSM types in V1M (orange, Fig. 8a). Since we generated a million macrolide scaffolds each containing 12 CSMs, the total number of CSMs used in the library was 12 million. Among all 12 million CSMs, a comparatively large portion in V1M were occupied by SM002 (methyl, 2.9 million, 24%) and SM001 (methylene, 2.2 million, 18%). The high occurrence for these SMs was obviously fueled by the high number of repetitions we initially allowed for this enumeration (SM002 was allowed four times, and SM001 three). The SM013 alkene was allowed two times and was comprised in 1.58 M (13%). Some CSMs containing oxygen such as SM004, SM005 and SM009 were comprised in relatively small portions (from 600 k to 800 k, 6%). The remaining SMs (SM003 containing an ethyl, SM006 containing an α-hydroxy methyl, and SM008 with a methyl carboxaldehyde) were comprised in ~ 1–1.2 M (9%) of the entire SM population. Regarding the eighteen BM scaffolds, a highly similar CSM type composition was observed (orange, Fig. 8b). Among a total of 163 CSMs found in eighteen BM scaffolds, SM002 (60, 36.8%) and SM001 (32, 19.6%) accounted for fairly large portions, as in V1M. The remaining seven CSM types among the BM scaffolds maintained a similar composition (4–8%) each. The direct comparison of CSM type composition (in percentages) among the eighteen BM scaffolds and V1M (Additional file 2: Figure S4B) emphasized a remarkably similar pattern, which was contributed by the carefully weighted inputs for SM type frequencies in generating V1M.

Regarding their occurrence per macrolide scaffold, we limited the occurrences of five CSM types: SM003, SM004, SM005, SM008, SM009, to only one per scaffold. Thus, it is not surprising to observe that their recurrences per macrolide scaffold in V1M were only one (Fig. 8c). Despite having different distributions for each repetition (which was not controlled during the enumeration process), V1M contained SM001, SM002, SM006 and SM013 up to their maximally allowed repetitions per macrolide scaffold (Fig. 8c). It demonstrates that the various user constraints are fully respected in the macrolide scaffold structures generated by the PKS Enumerator software. In comparison to the eighteen BM scaffolds (Fig. 8d), the frequency distributions of the CSMs in V1M appear more balanced or controlled (Fig. 8c); in other words, they form a slightly bell-shaped pattern. On the other hand, there is no recognizable pattern among the different occurrences of CSMs among the eighteen BM scaffolds.

**Fig. 9:**
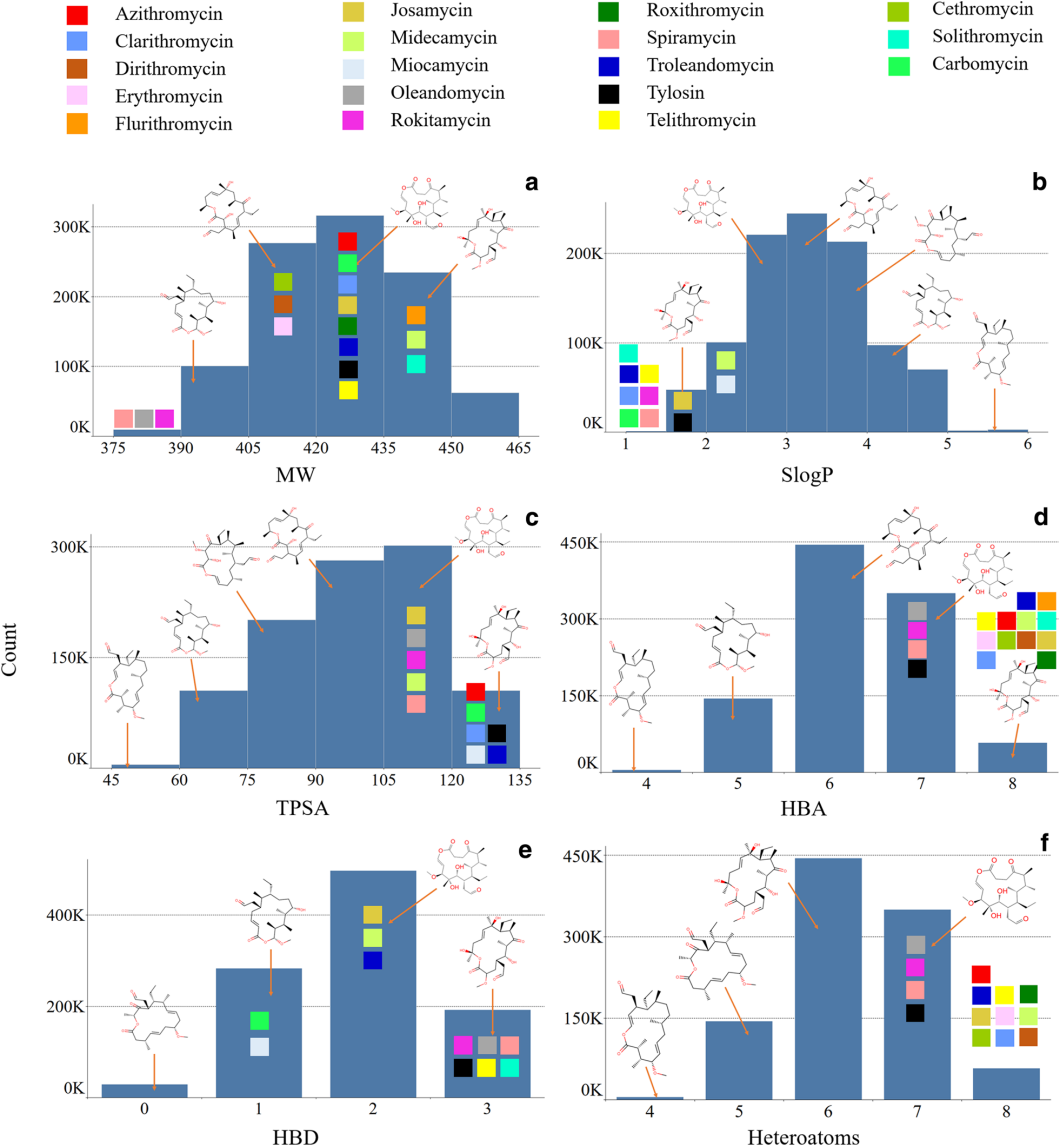
Distribution of molecular properties: **a** Molecular Weight–MW, **b** Hydrophobicity–SlogP, **c** Topological Polar Surface Area–TPSA, **d** Hydrogen Bond Acceptors–HBA, **e** Hydrogen Bond Donors–HBD, **f** Hetero Atoms of 1 million macrolide scaffolds in V1M. 2D-structures of randomly selected macrolides from V1M were displayed in associated bins of molecular properties. Eighteen BM scaffolds which fell within the range of molecular properties of V1M were also color-coded and displayed in associated bins

The number of O-heteroatoms, which is the only type of heteroatom in V1M solely based on the selected CSMs, ranged from 4 to 8 (Fig. 9f). The highest populations with approx. 444 k and 350 k macrolide scaffolds had six and seven heteroatoms, respectively, and 144 k macrocycles contained five O-heteroatoms. The diversity of heteroatoms in the library can be easily controlled by using RSMs that could introduce alternative heteroatoms other than oxygen, such as nitrogen, sulfur, phosphorus, boron, etc. However, we specifically chose commonly found CSMs derived from the eighteen BM scaffolds and excluded RSMs that are normally enriched with different heavy atoms and/or functional groups. In comparison to the eighteen BM scaffolds, which contained 7 to 9 heteroatoms, V1M delivered relatively lower numbers of heteroatoms. The number of heavy atoms in V1M followed a slightly left-skewed distribution ranging from 27 to 32 (Additional file 2: Figure S5B). Most of the library (858 k macrolide scaffolds) had 29 to 31 heavy atoms. A higher diversity in the number of heavy atoms can be delivered by adjusting the ring size or structural motifs with different lengths and/or functional groups. All BM scaffolds were composed of 27, 29, 30, 31 and 34 heavyatoms per ring, and seventeen BM scaffolds were observed within the heavyatom distribution of V1M.

### Chemical diversity of V1M

We analyzed the chemical diversity section of V1M library in terms of six molecular properties: molecular weight (MW), hydrophobicity (SlogP), topological polar surface area (TPSA), hydrogen bond acceptors (HBA), hydrogen bond donors (HBD), and rotatable bonds (NRB). These specific molecular properties were selected because they are commonly used to assess oral absorption, cell permeability, bioavailability, and drug likeness [38–43] of small molecules.

The molecular weight of V1M followed a slightly left-skewed distribution. It ranged from 378.5 to 456.6 g mol^−1^ with an average (mean_MW_) equal to 422.6 ± 16.7 g mol^−1^ (Fig. 9a). The highest population with approximately 316 k macrolide scaffolds (31.6%) fell between 420 and 435 g mol^−1^. Remarkably, 828 k (83%) of V1M population fell within the narrow range of MW from 405 to 450 g mol^−1^, along with seventeen out of the eighteen BM scaffolds which ranged from 384.22 to 482.25 g mol^−1^ with an average of 424.7 ± 23.7 g mol^−1^. Regarding Lipinski’s rule of 5 [39], V1M and eighteen BM scaffolds abided by Lipinski’s molecular weight since they all had MW less than 500. However, the original structures of eighteen BMs were simplified by removing sugars and bulky functional groups for a direct comparative study with V1M. Even after the removal of commonly occurring sugar groups which would amount to approx. 320 g mol^−1^ and bulky functional groups, eighteen BM scaffolds were found to have MWs very close to the MW threshold. Therefore, based on the eighteen bioactive macrolide scaffolds we studied, MW limit of 500 g mol^−1^ may not be quite relevant for macrolides.

Then, we analyzed the distribution of the predicted hydrophobicity as assessed by the fragment-based octanol/water coefficient partition (SlogP) for all generated macrolide scaffolds in V1M. SlogP had a bell-shaped symmetric distribution with values ranging from 1.19 to 5.57, and an average of 3.29 ± 0.77 **(**Fig. 9b). The highest population with approximately 244 k macrolide scaffolds (24.48%) fell in the SlogP range between 3 and 3.5. A very small percentage (0.48%) of V1M had SlogP > 5, thereby exceeding Lipinski’s rule regarding hydrophobicity. On the other hand, SlogP of eighteen BM scaffolds abided by Lipinski’s rule; they ranged from 0.17 to 2.69 with an average of 1.17 ± 0.65. All eighteen BM scaffolds were observed either below or within the low spectrum of SlogP distribution in V1M, suggesting that low/moderate hydrophobicity is preferred for potent antibiotic macrolide drugs.

Topological Polar Surface Area (TPSA) appeared to follow a left-skewed distribution. It ranged from 52.6 to 130.4 Å^2^ (Fig. 9c) with an average of 99.5 ± 15.6 Å^2^. Approximately 302 k (30.2%) fell in the most populated region between 105 and 120 Å^2^, and 99.5% of the library had TPSA between 60 and 135, in which 11 BM scaffolds (61.1%) were observed as well. TPSA of the eighteen BM scaffolds ranging from 113 to 153 Å^2^ were observed in the high end and beyond the maximum range of V1M. Since macrolides are known for their ability to bind difficult target proteins with bland surfaces and large binding pockets [9], higher TPSAs observed for these eighteen BMs could be highly relevant to their potent bioactivities. Overall, both V1M had thirteen BM scaffolds had TPSAs compliant with Veber’s rules [38], TPSA ≤ 140 Å^2^ beyond which five BM scaffolds were observed.

The number of hydrogen bond acceptors (HBA) in V1M followed a slightly right-skewed distribution (Fig. 9d). V1M ranged from 4 to 8 HBAs with an average of 6.31 ± 0.8. Approximately 938 k macrolide scaffolds (93.8%) of V1M was observed to have HBAs from 5 to 7, and a majority 444 k (44.4%) macrolide scaffolds had 6 HBA. However, higher range of HBA (7 to 9) was observed for all BM scaffolds, suggesting a higher count of HBAs could be relevant to the potent bioactivities of macrolides. Both V1M and eighteen BM scaffolds were compliant with Lipinski’s rule regarding HBA (HBAs ≤ 10). Meanwhile, the numbers of hydrogen bond donors in V1M ranged from 0 to 3, with 497 k (49.7%) macrolide scaffolds have 2 HBDs (Fig. 9e). BM scaffolds covered a wide range of HBDs from 1 to 6, with 11 BM scaffolds within HBD distribution of V1M. Sixteen BM scaffolds and V1M had HBD values compliant with Lipinski’s rule (HBD ≤ 5). Two BM scaffolds had HBDs of 6. The number of rotatable bonds in each macrolide scaffold ranged from three to four (Additional file 2: Figure S5A). A large majority, approximately 800 k (80%), of V1M had rotatable bonds of four. All BM scaffolds covered a range of rotatable bonds from 0 to 6 with an average of 2.33 ± 1.68. Seven BM scaffolds had NRB of 1, and only five BM scaffolds fell within the same distribution of V1M. Both our V1M library and 18 BM scaffolds were compliant with Veber’s rule of NRB (NRB ≤ 10).

Since we have been referencing Lipinski’s rule of 5 and Veber’s rules for our macrolide scaffolds, we also conducted a short study to assess whether the eighteen chosen BMs with reduced structures (Additional file 2: Figure S2) followed these rules as well. Additional file 2: Figure S6 shows a summary of molecular properties and filters such as MW, SlogP, TPSA, HBA, HBD, NRB that are normally used to determine cell permeability, bioavailability and drug likeness [38, 40, 41], along with color-coded information on whether the molecular property values fall within or outside Lipinski’s and Veber’s region.

Thirteen out of eighteen BM scaffolds displayed molecular properties well within Lipinski’s [39] and Veber’s rules[38] while the rest slightly deviate in TPSA and HBD (Additional file 2: Figure S6). All BM scaffolds display MW ≤ , SlogP ≤ 5, HBA ≤ 11 and NRB ≤ 10. Five deviating BM scaffolds still showed values not far from the Lipinski’s and Veber’s marginal values (HBD = 5, TPSA = 140). Dirithromycin (HBD = 6, TPSA = 153.47) and Roxithromycin (HBD = 6, TPSA = 151.3) showed the highest deviations from the Lipinski’s border values of HBD while the rest three BM scaffolds (Cethromycin, Erythromycin and Flurithromycin) slightly deviated from Veber’s TPSA of 140 by a range of 4.52–7.15. This data suggested that, as expected, not all BM scaffolds abided by Lipinski’s or Veber’s rules [39], but the majority of BM scaffolds still fell within Lipinski’s region of drug likeness and bioavailability. One should underscore again that our analysis was conducted using the reduced representation of the BMs (i.e., only the macrolide scaffolds) to enable the direct comparison with the macrolide scaffolds generated by the PKS Enumerator. One should also underline that estimating the drug likeness of macrolides is highly complex; therefore, rules derived from small aliphatic molecules are likely to fail.

### Correlation analysis among computed descriptors of V1M

To better understand the relationships among the chemical descriptors, we analyzed the Pearson correlation coefficients among all molecular properties (MW, SlogP, TPSA, HBA, HBD, heteroatoms and heavyatoms) computed for V1M. The heatmap is reported in Additional file 2: Figure S7. Several interesting patterns emerged during this pairwise correlation analysis among descriptors. TPSA was observed to hold multiple strong positive relationships with other molecular descriptors except for SlogP and rotatable bonds. TPSA established strong positive correlations with HBA (r = 0.97), HBD (r = 0.88), and heteroatoms (r = 0.97). Predictably, introducing heteroatoms, especially polar atoms such as oxygen or nitrogen or fluorine, would increase polar surface areas and promote hydrogen bonding as well. The chemical descriptors related to polarity and hydrogen bonding (TPSA, HBA, HBD and heteroatoms) all had strong positive correlations among each other; and some more than the others. For example, HBA established a perfect positive correlation with heteroatoms. Oxygen was the only heteroatom introduced in CSMs (SM004, SM005, SM006, SM008 and SM009) used for our study, thus it is the major source affecting important chemical properties which are TPSA, HBA and HBD. Introducing polar functional groups by carefully designing new SM types, selecting and specifying the occurrences of SM types per macrolide scaffold could have a significant impact on the associated chemical properties.

MW had an unsurprisingly strong positive correlation with heavyatoms (r = 0.99). MW also showed relatively strong positive correlations with HBA (r = 0.71), HBD (r = 0.67), TPSA (r = 0.66). It was likely because the CSM types containing oxygen had relatively larger functional groups in comparison to the rest in our study (e.g. SM005, SM006, SM008), thereby resulting in a high correlation between MW and other descriptors: TPSA, HBA and HBD. This correlation can be enhanced by allowing higher number of CSMs with polar atoms in the selection pool or increasing their repeats per macrolide scaffold. In general, it can be seen in V1M that several molecular properties such as MW, TPSA, HBA, HBD, heavyatoms and heteroatoms had moderate or strong positive correlations with one another.

Comprehensibly, SlogP (hydrophobicity) established multiple strong negative relationships with other molecular descriptors: TPSA (r = − 0.94), HBA (r = − 0.93), HBD (r = − 0.77), and heteroatoms (r = − 0.93). Since most polar compounds are known to interact with water, lower hydrophobicity would be observed for macrolides possessing larger polar surface areas or functional groups with potential HBAs and HBDs. SlogP didn’t show good correlations with MW (r = − 0.44) and NRB (r = 0.04). NRB did not report any important correlations with the rest of the molecular descriptors, and the distribution of NRB within V1M (Additional file 2: Figure S5A) was too low to form any significant correlations with other descriptors.

### Analysis of chemical fingerprints for V1M

Chemical fingerprints were computed for the scaffolds of both V1M and the eighteen BMs. We used 2D MACCS (RDKit implementation of the MACCS keys [36]) via the RDKit fingerprint node in Knime. Tanimoto similarity coefficients were computed via the CDK toolkit in Knime for the fingerprints of V1M against those of eighteen BM scaffolds which were used as reference compounds. For each of 1 M macrolide scaffolds in V1M, only the maximum Tanimoto score achieved with any of the eighteen BM scaffolds was reported, i.e., maximum aggregation method. For example, a macrolide scaffold would afford various Tanimoto scores with all the eighteen BM scaffolds, among which it afforded the highest Tanimoto score with Clarithromycin. So, for that macrolide scaffold, only Clarithromycin and the associated Tanimoto score was reported. We then analyzed the distribution of Tanimoto scores obtained for all 1 M macrolide scaffolds. Tanimoto scores range from 0 to 1, with 1 being the highest similarity score between two compounds and 0 the lowest. It should be noted that the Tanimoto score between Spiramycin and Rokitamycin is 1 (Additional file 2: Figure S8), which would explain why macrolide scaffolds in V1M obtained the same Tanimoto scores with both. It should also be noted that MACCS method does not account for chirality since they are 2D fingerprints based; thus, there is a clear limitation in determining chemical similarity for compounds with different stereocenters.

**Fig. 10:**
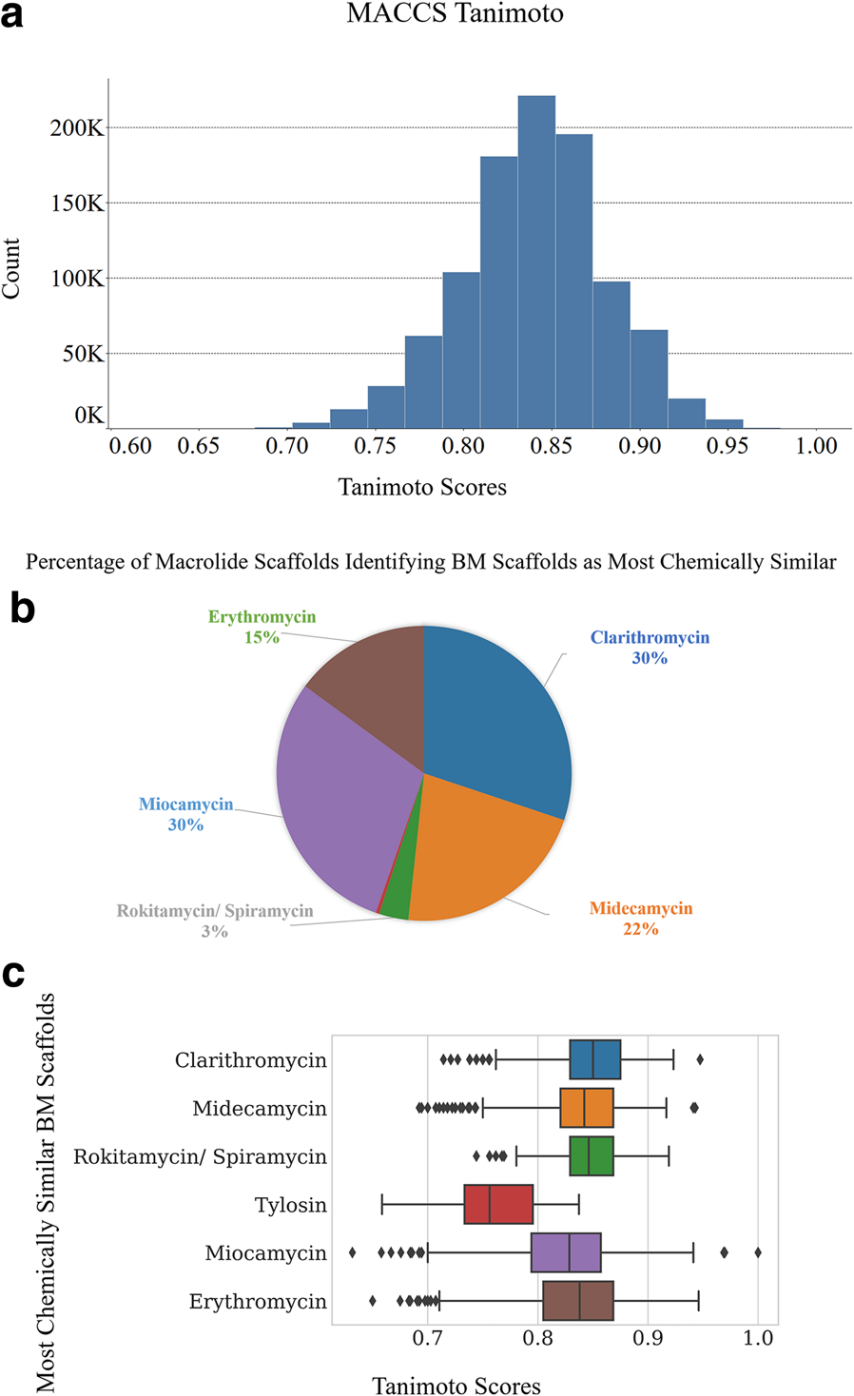
Calculation of Tanimoto similarity scores between V1M and the 18 BM scaffolds as probe molecules using MACCS Fingerprint and maximum aggregation method. **a** Highest Tanimoto distribution of V1M. Number of macrolide scaffolds in V1M identifying eight BM scaffolds as highest chemically similar; **b** pie chart with count of macrolide scaffolds reported as percentage of the population; **c** box plot analysis of eight BM scaffolds with associated Tanimoto score distributions

V1M had a slightly left-skewed distribution of MACCS Tanimoto scores ranging from 0.63 to 1.0, with an average of 0.84 ± 0.04 (Fig. 10a). The macrolide scaffolds in V1M identified seven among the eighteen BM scaffolds as most chemically similar: Clarithromycin, Midecamycin, Rokitamycin, Spiramycin, Tylosin, MiocaV1Mmycin and Erythromycin (Fig. 10b).

The count of macrolide scaffolds with highest Tanimoto scores associated with these seven BM scaffolds is reported in Fig. 10b. A large portion of V1M, 297 k (30%) and 301 k macrolide scaffolds (30%), was associated with the highest MACCS-based chemical similarity with Miocamycin and Clarithromycin respectively among all other BM scaffolds (Fig. 10b). Approximately one quarter of V1M, 216 k (22%) macrolide scaffolds, identified Midecamycin, and 149 k macrolide scaffolds (15%) identified Erythromycin as the most chemically similar BM scaffolds. Only 32 k macrolide scaffolds (3%) identified Rokitamycin/Spiramycin, and 3.9 k macrolide scaffolds (0.39%) Tylosin as the highest chemically similar BM scaffold.

**Fig. 11:**
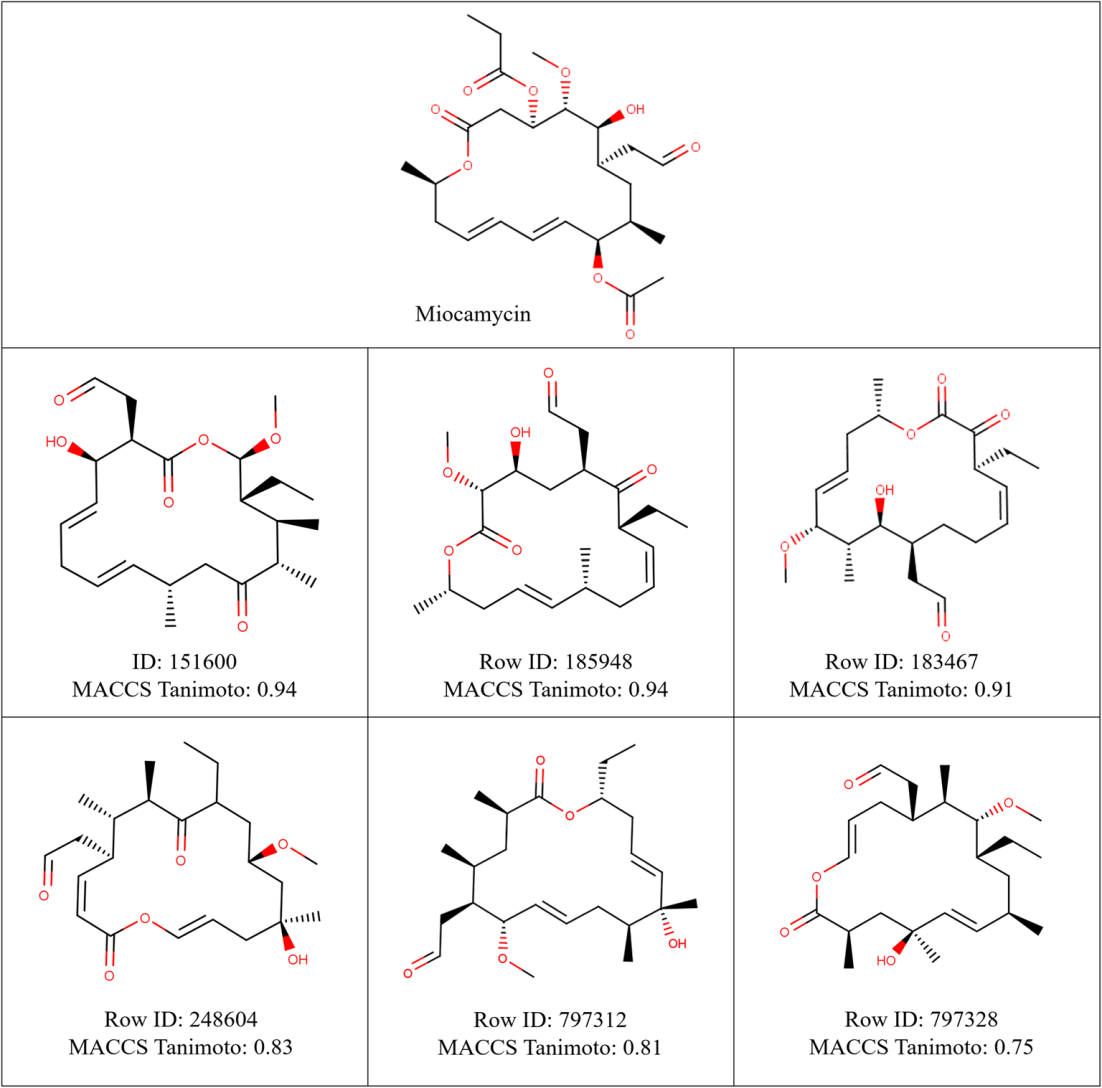
Example macrolide scaffolds from V1M identified as close analogues of Miocamycin, based on their Tanimoto coefficient and MACCS fingerprints

The boxplot analysis in Fig. 10c showed the distributions of Tanimoto scores against these seven BM scaffolds. This allowed us to determine the level of chemical similarity between individual BM scaffolds identified as most chemically similar, and their closest macrolide scaffold analogues in V1M. Clarithromycin (0.853 ± 0.03), Midecamycin (0.846 ± 0.03) and Rokitamycin/Spiramycin (0.849 ± 0.03) showed similar distributions with a median Tanimoto score of approximatively 0.85 (Fig. 10c), indicating that macrolide scaffolds in V1M afforded an equivalent level of chemical similarity with these BM scaffolds. Miocamycin (0.826 ± 0.05) and Erythromycin (0.831 ± 0.04) covered relatively wider, but somewhat similar Tanimoto distributions with a median Tanimoto score of approximately 0.83 (Fig. 10c). Overall, their closest analogues from V1M showed an equivalent level of chemical similarity with these BM scaffolds; except for Tylosin which had relatively lower Tanimoto scores ranging from 0.66 to 0.84 with an average of 0.76 ± 0.03. Among the macrolide scaffolds in V1M that had highest fingerprint similarity with Miocamycin, six example structures along with their Tanimoto scores were provided in Fig. 11. Using Tanimoto score of 0.75 as a cutoff value for good similarity measurement, 987 k macrolide scaffolds in V1M achieved high chemical similarity with the known BM scaffolds based on MACCS fingerprint.

## Discussion

One of our primary goals in this study was to develop a method and associated software capable of efficiently generating very large libraries of virtual screening-ready macrolide scaffolds that include biosynthetically-amenable building blocks found in known bioactive macrolides. That is why we studied eighteen known bioactive macrolide drugs (Additional file 2: Figure S1) and included only the CSMs (the common structural motifs found in at least five among the eighteen BMs) to generate V1M library, which has been shown to contain analogues with high structural similarity towards the known bioactive macrolides. We posit it is possible to synthesize many of these enumerated virtual macrolide scaffolds using the combinatorial biosynthesis technology which applies synthetic biology concepts to engineer polyketide synthase modules, mentioned in the “Introduction” section. Additionally, with the surge of powerful technologies, supercomputers and smart algorithms, there is much hope in synthesizing such complex and innovative compounds using AI-powered retrosynthetic route planning software (e.g., Chematica [44]).

However, it should be noted that using the derived building blocks from known bioactive macrolides or sharing strong chemical similarity with the already-existing analogues do not guarantee stable or experimentally synthesizable compounds, since certain arrangements of SMs can result in unstable chemical components. For example, functional groups such as hydroxymethylester (CO–O–C–OH) or methoxymethylester (CO–O–C–OMe) are unstable and perhaps impossible to form and they do not occur in any of the bioactive macrolides we studied. The technology of our PKS Enumerator software does not address synthetic feasibility, stability, or toxicity of the enumerated compounds since each feature, being a project worthy of a research paper on its own, involves a large scope of work. In the future, we aim to implement some of those useful additional features to enhance the capabilities of PKS Enumerator.

According to Lipinski’s rule of 5, compounds with MW > 500, SlogP > 5, HBD > 5 and HBA > 10 show low potential for druglikeness due to poor oral absorption [39]. Many bioactive compounds display Lipinski’s characteristics [43], and certain drug classes, including antibiotic macrolides, have been known for drug-like potential including reasonable absorption, cell membrane permeability, and bioavailability [41, 43]. Additionally, filters such as topological polar surface area (≤ 140) [40, 41] and the number of rotatable bonds (≤ 10) [38, 41] are also good indicators of oral bioavailability. In V1M, all generated compounds displayed molecular properties well within Lipinski’s and Veber’s rules in terms of MW, TPSA, HBA, HBD, and NRB, and a large majority of V1M (99.5%) displayed SlogP lower than 5.

Nonetheless, most bioactive macrolides violate Lipinski’s rules (e.g., macrolides with MW > 500) but are still bioactive and afford reasonable bioavailability [10]. Therefore, strict reliance on those rules could be a hindrance in exploring the chemical space of macrolides for novel antibiotics due to their unique structural features and biological properties [39, 41, 42]. One way to approach macrolides would be to establish predictive (Q)SAR models from bioactive compounds and explore how their unique structural features contribute to their potencies. It would certainly give us more insight for developing new compounds.

The analysis part of the manuscript may appear to some readers as either “obvious” or “common sense”. However, there is a plethora of benefits to gain from such exploratory and descriptive statistics. The structural and chemical diversity analyses further reflect the depth of control users can have in designing and manipulating these virtual chemical libraries. Chemical similarity statistics can be used to finetune the libraries: researchers can create either highly-focused set of compounds sharing strong chemical similarity towards the target molecules, or diverse set of compounds with different building blocks and molecular descriptors.

There are several types of applications we plan to accomplish with this enumeration technology. We aim to generate libraries of macrocycles with very specific molecular properties, and thus create highly-focused sets of compounds, not just structure-wise, but also property-wise. In other words, we can provide a range for each molecular descriptor (*e.g.,* MW, SlogP, TPSA, HBA, HBD, NRB) and the enumerator will select only the macrocycles with molecular properties that fall within user-defined ranges. This could be done in addition to similarity constraints toward a particular active macrolide probe. Currently, that step is done post-enumeration. Moreover, we will virtually screen PKS Enumerator library using molecular docking to test tens of millions of diverse macrolide scaffolds against several biological targets of relevance. These molecular docking findings will be used to prioritize new macrolide biosynthesis and potential experimental testing.

The macrolide scaffolds generated by our PKS Enumerator lack sugar components found in bioactive macrolides. More research is needed to correctly and systematically incorporate sugars into our macrolide scaffolds. For example, how do we determine all the potential positions in a given macrolide scaffold? Would sugars be attached to the same SMs throughout the entire library? If not, how should we implement it as an option in our software? What if the chosen SMs are not always part of the macrolide scaffolds? Or if the chosen SMs can repeat twice or more, to which SM(s) should we attach sugars? Should sugars be added pre- or post- enumeration? We plan to resolve these questions and include the corresponding feature in a future version of our software.

At last, we compared our PKS Enumerator to BoBER (web server Base of Bioisosterically Exchangeable Replacements) [35]. The purpose and approach behind these cheminformatic tools are very different. The purpose of PKS Enumerator is to diversify the class of macrocycles and macrolides by adding structural motifs one by one and permuting them, while offering the option to introduce novel structural motifs into macrocycle/macrolide scaffolds. On the other hand, BoBER aims to improve activity, reduce toxicity, change bioavailability using the concept of bioisosterism and scaffold hopping [35]. These two cheminformatics tools, however, can be complementary to one another such that some structural fragments from macrocycle/macrolide scaffolds generated by PKS Enumerator can be replaced with other fragments using BoBER to improve overall biological activities and pharmacokinetic properties.

## Conclusion

V1M, the virtual library of 1 million macrolide scaffolds created in this study using our PKS Enumerator software, is the largest publicly accessible library of macrolide scaffolds. This library including chemical structures, and computed properties is provided as supplemental material. We showed that modern molecular enumeration software enables the computationally-efficient generation of diversity-controlled and extremely large chemical libraries of macrolide scaffolds with well-defined structural constraints. Such new type of enumeration technology diversifies the class of macrolides by offering a plethora of innovative and unexplored structures. Hence, it widens the scope of macrolides to motivate the search of polyketide drugs for design and synthesis, and consequently helps advance pharmaceutical development. Chemical diversity analysis of V1M showed well-distributed molecular properties of interest, and druglike characteristics (based on Lipinski’s and Veber’s rules) for a vast majority of 1 million macrolide scaffolds. Importantly, V1M contained analogues that share high chemical similarity with well-known bioactive macrolides. This is certainly hopeful for future studies in search of novel bioactive macrolides.

## Additional files

**Additional file 1.** Chemical structures of V1M macrolide scaffolds.

**Additional file 2.** **Table S1**: Common Structural Motif (CSM) Type Distribution and Occurrence per Macrolide Scaffold among Eighteen Bioactive Macrolide (BM) Scaffolds. **Figure S1**: Sixteen structural motifs (nine CSMs and seven RSMs) currently employed in PKS Enumerator software, along with the bioactive macrolides from which they were derived.** Figure S2**: Modified structures of eighteen well-known bioactive macrolides (BMs). **Figure S3**: Structural simplification of Erythromycin for a comparative study with enumerated virtual macrolide scaffolds from V1M. **Figure S4**: Percentage of (A) macrolide scaffolds in which associated CSM types were found, and (B) CSM type composition, in 18 BMs and V1M. **Figure S5**: Distribution of (A) rotatable bonds, and (B) heavy atoms in V1M. **Figure S6**: Color-coded map to demonstrate the molecular properties of eighteen bioactive macrolide scaffolds in correlation to Lipinski’s and Veber’s rules. **Figure S7**: Pearson’s pair-wise correlation heatmap of all eight molecular descriptors of V1M library: MW – molecular weight, SlogP – hydrophobicity, TPSA - topological polar surface area, HBA – hydrogen bond acceptors, HBD – hydrogen bond donors, NRB – rotatable bonds, heteroatoms, heavy atoms. **Figure S8**: Modified structures of Rokitamycin and Spiramycin. The computed Tanimoto score between these two structures is 1, based on MACCS fingerprint method.

## Abbreviations

PKS: polyketides
V1M: virtual 1 million macrolide scaffolds
SM: structural motifs
CSM: common structural motifs
RSM: rare structural motifs
MW: molecular weight
SlogP: hydrophobicity
TPSA: topological polar surface area
HBA: hydrogen bond acceptors
HBD: hydrogen bond donors
NRB: number of rotatable bonds
