# Supplementary material for: Cheminformatics-based enumeration and analysis of large libraries of macrolide scaffolds
Source: J Cheminform. 2018 Nov 12;10:53. doi: 10.1186/s13321-018-0307-6 (PMC6755550; doi:10.1186/s13321-018-0307-6)
Supplement: Supplementary file 2 — Additional file 2. Table S1: Common Structural Motif (CSM) Type Distribution and Occurrence per Macrolide Scaffold among Eighteen Bioactive Macrolide (BM) Scaffolds. Figure S1: Sixteen structural motifs (nine CSMs and seven RSMs) currently employed in PKS Enumerator software, along with the bioactive macrolides from which they were derived. Figure S2: Modified structures of eighteen well-known bioactive macrolides (BMs). Figure S3: Structural simplification of Erythromycin for a comparative study with enumerated virtual macrolide scaffolds from V1M. Figure S4: Percentage of (A) macrolide scaffolds in which associated CSM types were found, and (B) CSM type composition, in 18 BMs and V1M. Figure S5: Distribution of (A) rotatable bonds, and (B) heavy atoms in V1M. Figure S6: Color-coded map to demonstrate the molecular properties of eighteen bioactive macrolide scaffolds in correlation to Lipinski’s and Veber’s rules. Figure S7: Pearson’s pair-wise correlation heatmap of all eight molecular descriptors of V1M library: MW – molecular weight, SlogP – hydrophobicity, TPSA - topological polar surface area, HBA – hydrogen bond acceptors, HBD – hydrogen bond donors, NRB – rotatable bonds, heteroatoms, heavy atoms. Figure S8: Modified structures of Rokitamycin and Spiramycin. The computed Tanimoto score between these two structures is 1, based on MACCS fingerprint method. [file 13321_2018_307_MOESM2_ESM.docx]

**Supplementary Material**

**Cheminformatics-based Enumeration and Analysis of**

**Large Libraries of Macrolide Scaffolds**

*Phyo Phyo Kyaw Zin^1,2^, Gavin Williams^1,3^, and Denis Fourches^1,2,3^**

^1^ Department of Chemistry, North Carolina State University, Raleigh, NC, USA.

^2^ Bioinformatics Research Center, North Carolina State University, Raleigh, NC, USA.

^3^ Comparative Medicine Institute, North Carolina State University, Raleigh, NC, USA.

* To whom correspondence should be sent. Email: [dfourch@ncsu.edu](mailto:dfourch@ncsu.edu)

**Table S1**. Common Structural Motif (CSM) Type Distribution and Occurrence per Macrolide Scaffold among Eighteen Bioactive Macrolide (BM) Scaffolds. The list of eighteen BMs are provided in **Figure 3** of the paper.

# Bioactive Macrolides – the number of bioactive macrolide scaffolds in which corresponding CSM types were found. # CSM Occurrence per Macrolide Scaffold – the repetition of corresponding CSM type observed in each macrolide scaffold, reported in ranges as it varied across different macrolide scaffolds.


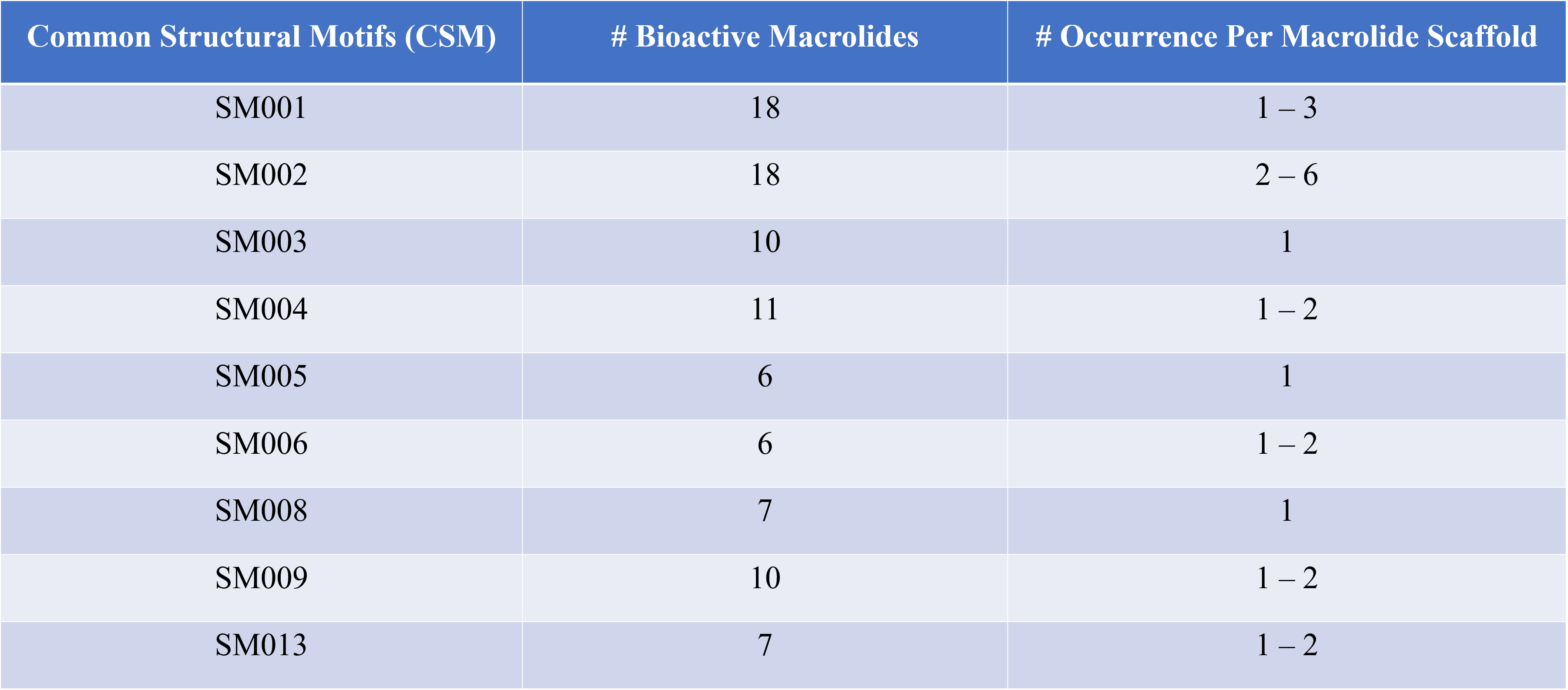


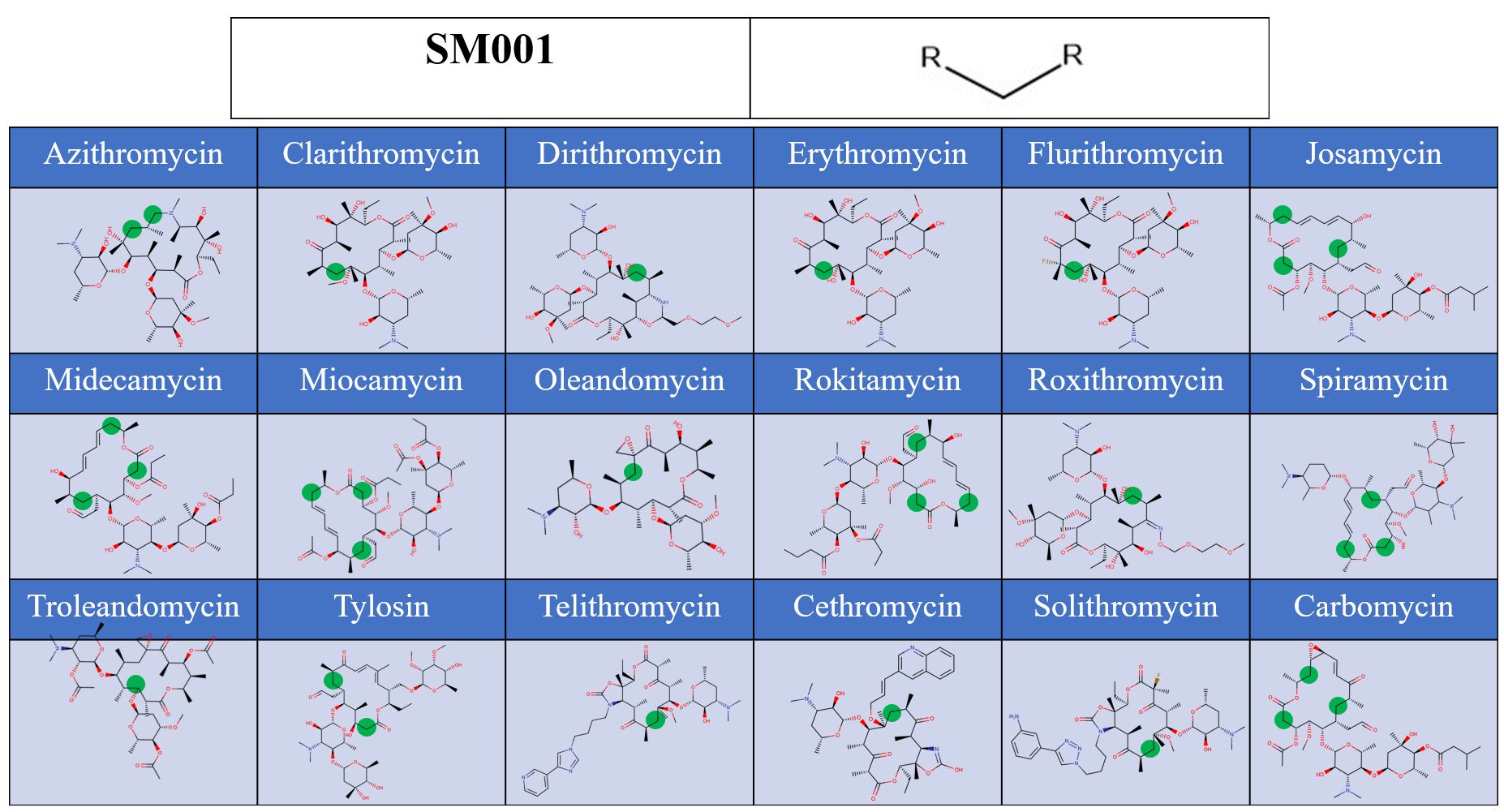


**Figure S1A**. SM001 (methylene) found and derived from bioactive macrolide scaffolds


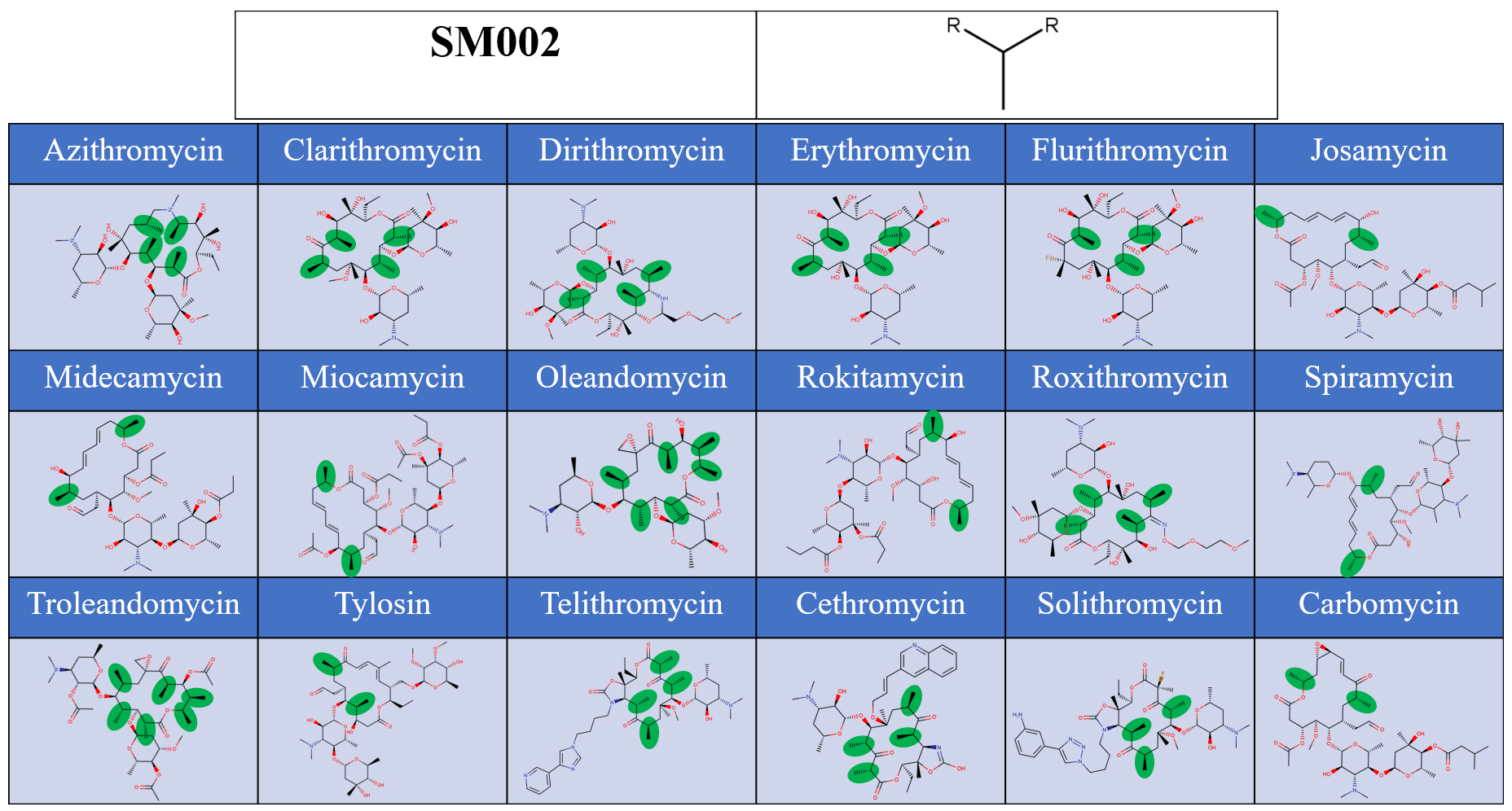


**Figure S1B**. SM002 (methyl) found and derived from bioactive macrolide scaffolds


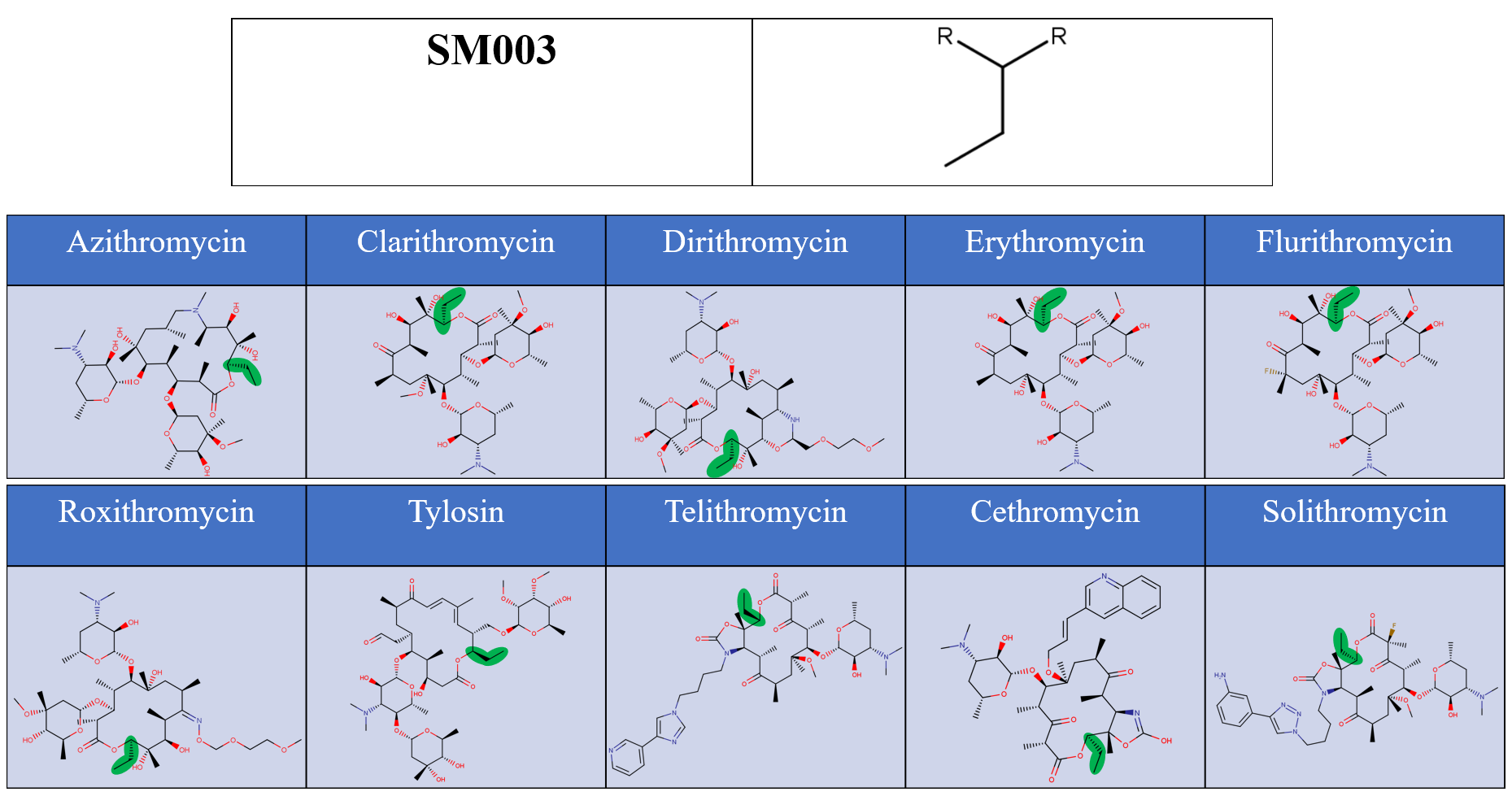


**Figure S1C**. SM003 (ethyl) found and derived from bioactive macrolide scaffolds


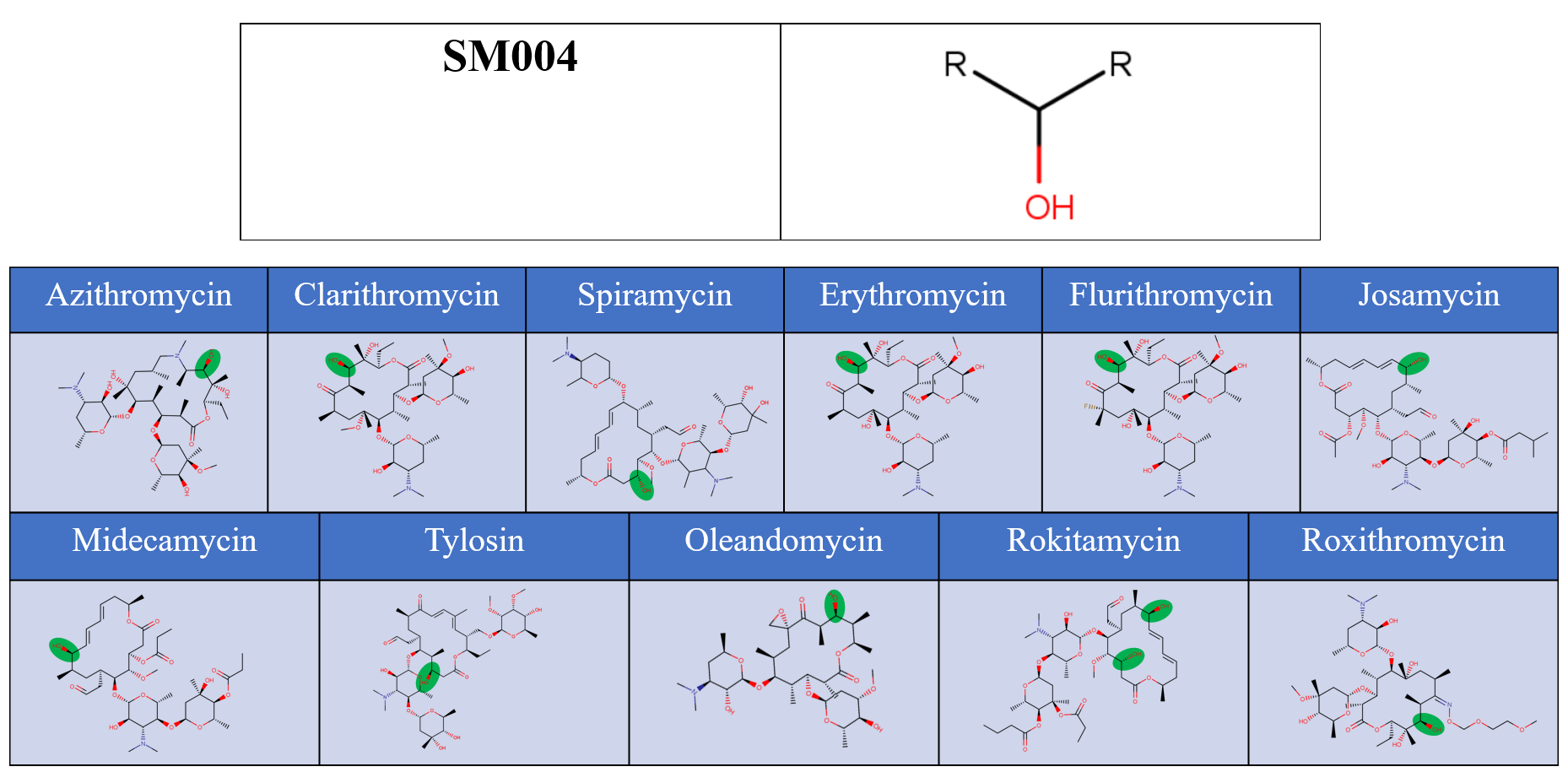


**Figure S1D**. SM004 (hydroxy) found and derived from bioactive macrolide scaffolds


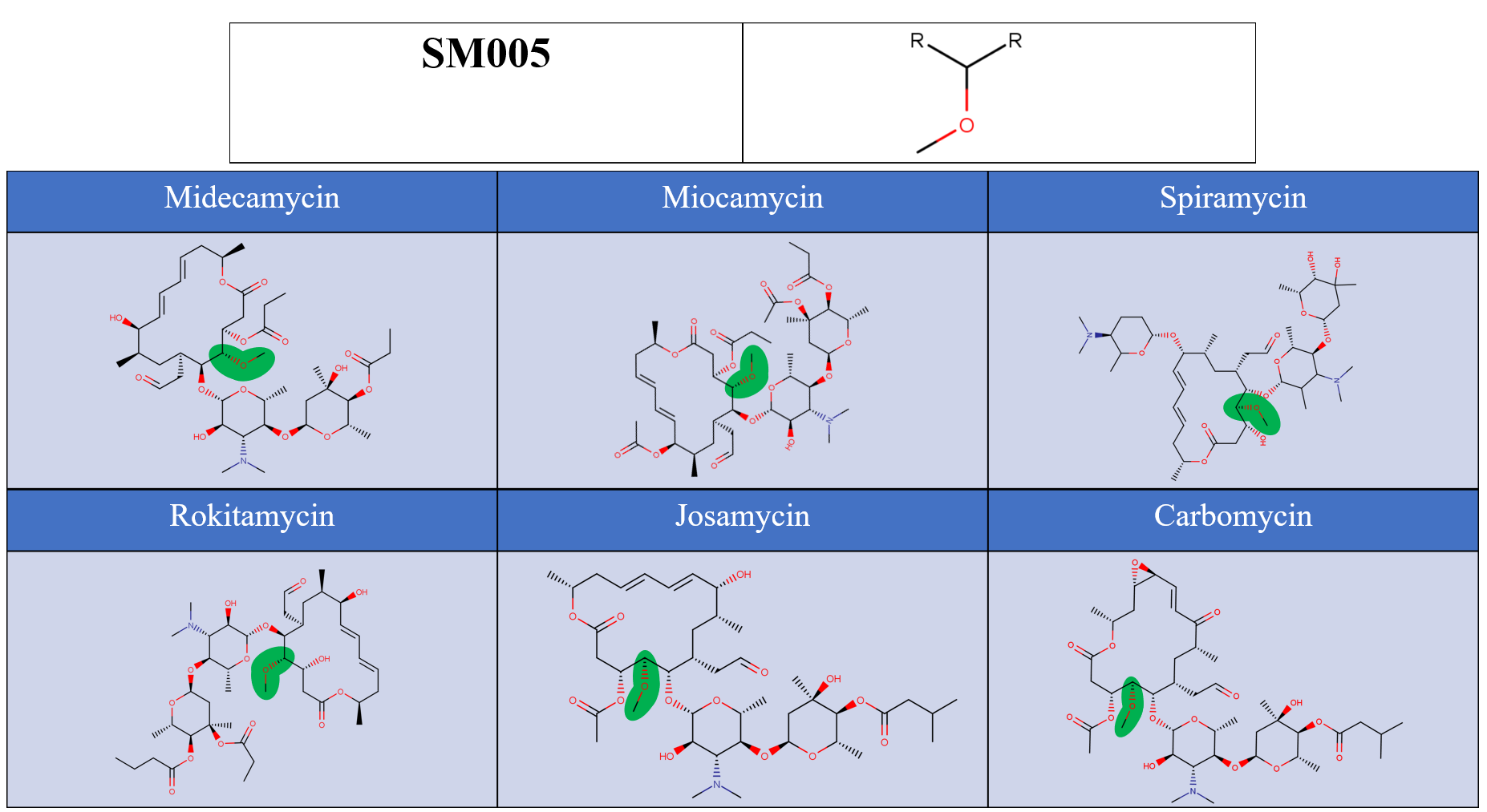


**Figure S1E.** SM005 (methoxy) found and derived from bioactive macrolide scaffolds


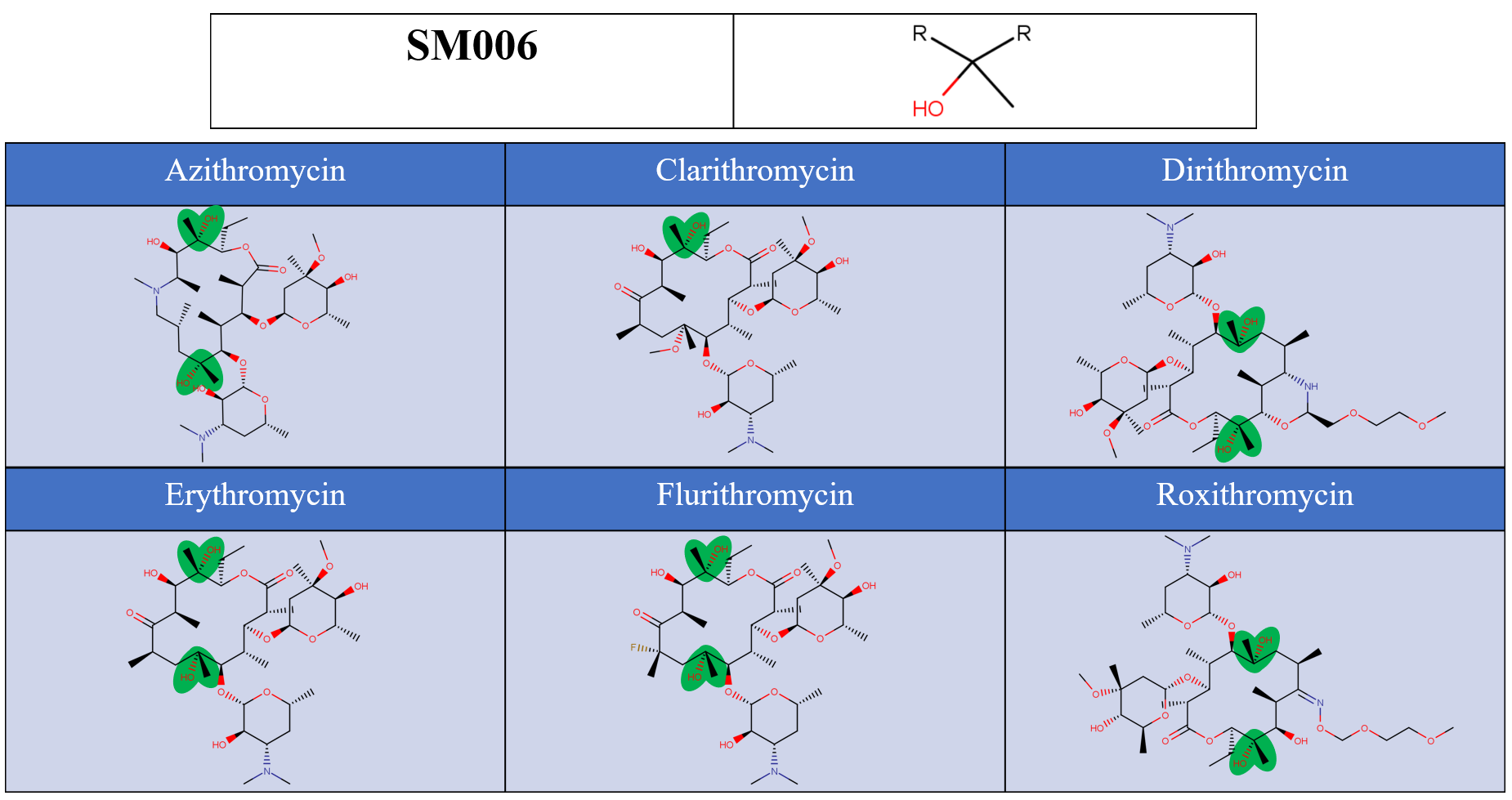


**Figure S1F.** SM006 (α-hydroxy methyl) found and derived from bioactive macrolide scaffolds


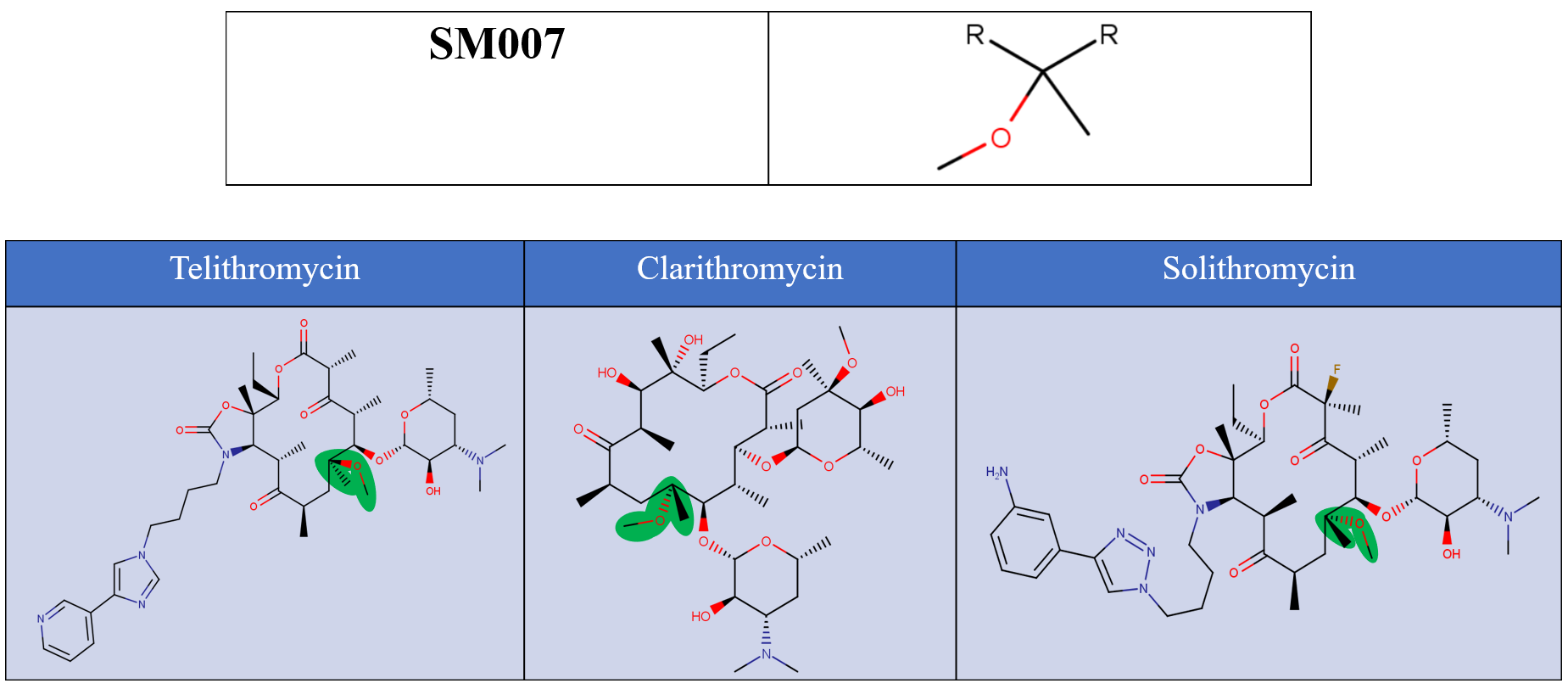
**Figure S1G.** SM007 (α-methoxy methyl) found and derived from bioactive macrolide scaffolds


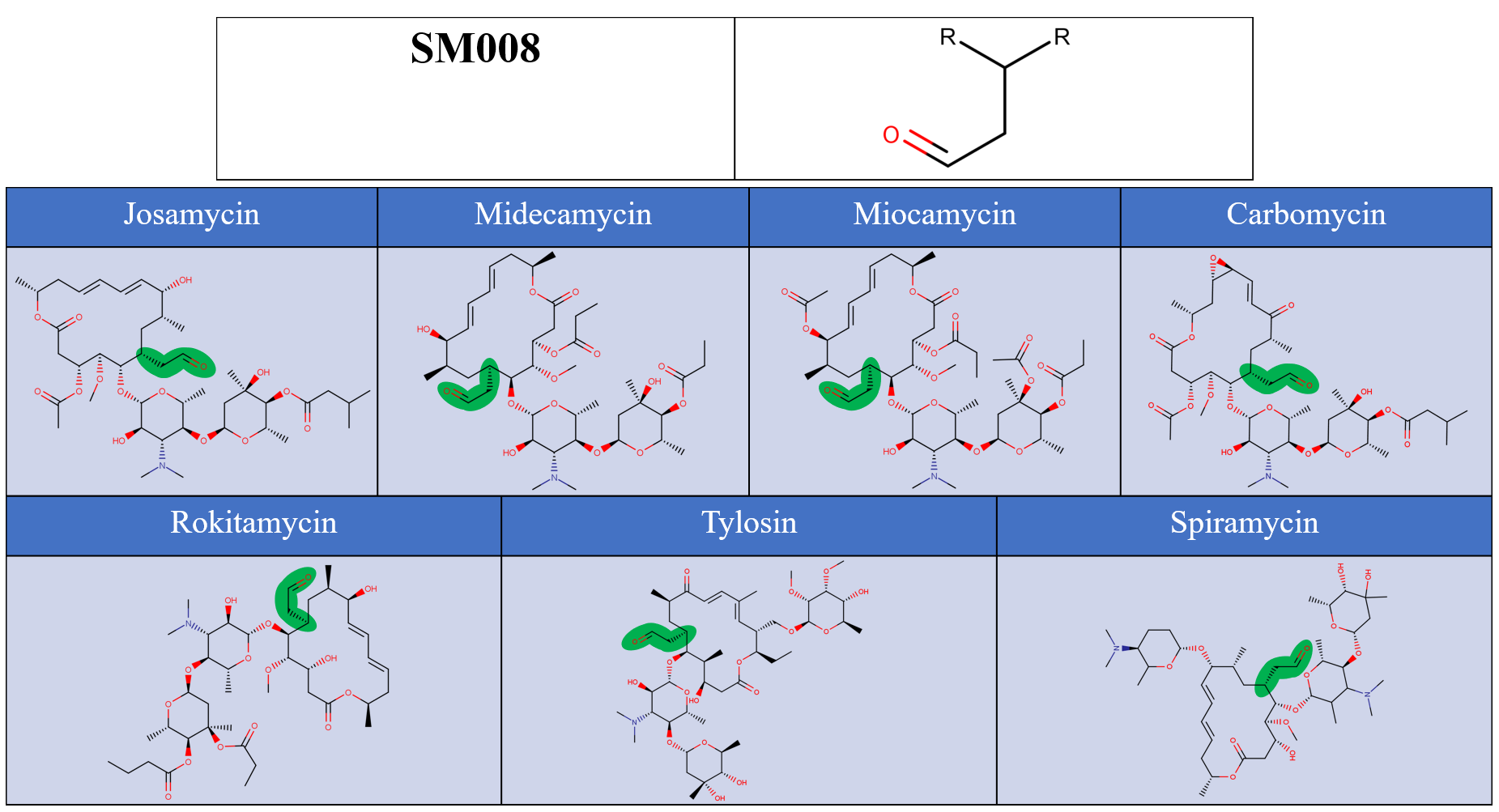


**Figure S1H.** SM008 (methyl carboxaldehyde) found and derived from bioactive macrolide scaffolds


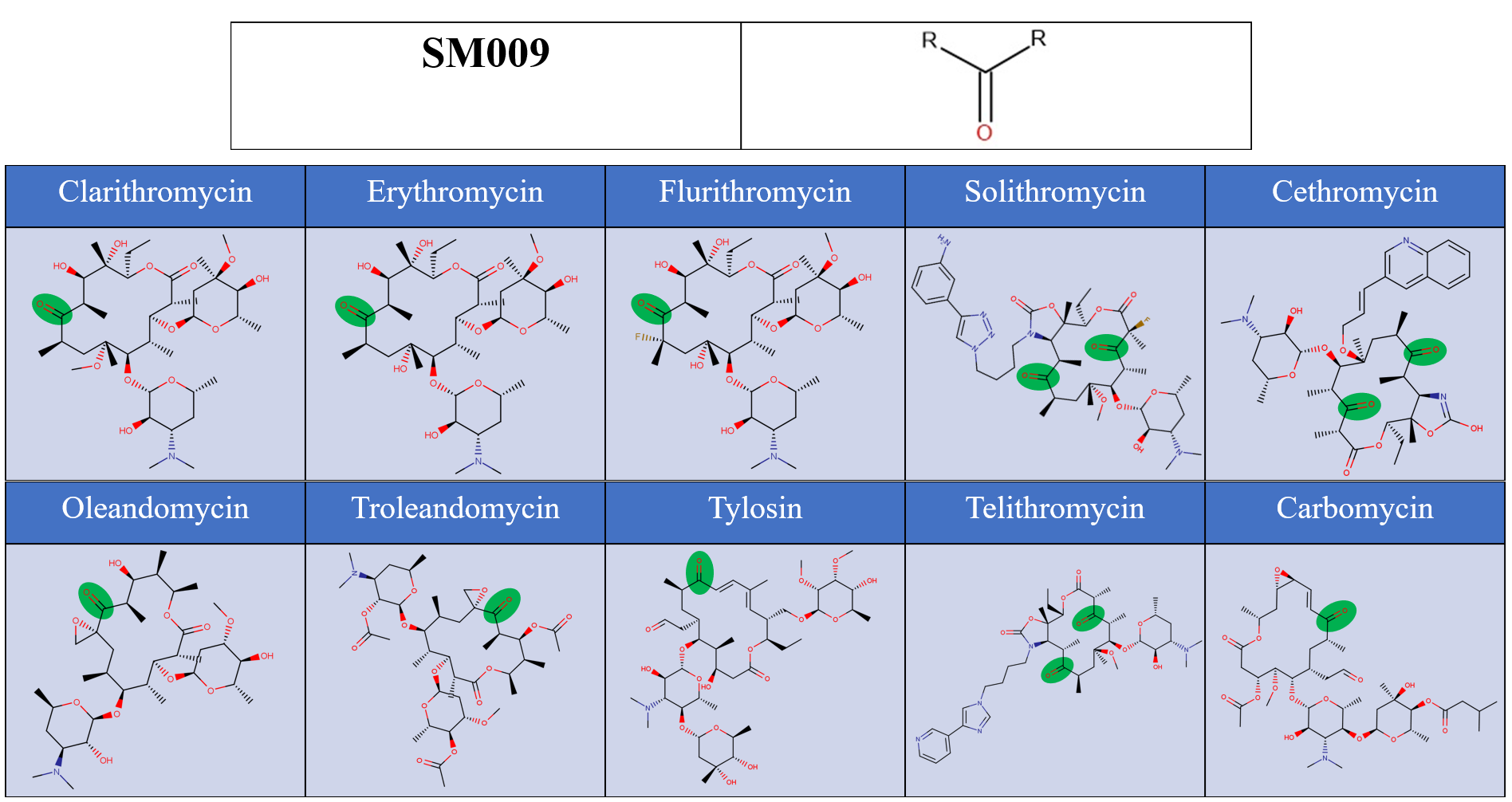
**Figure S1I.** SM009 (keto) found and derived from bioactive macrolide scaffolds


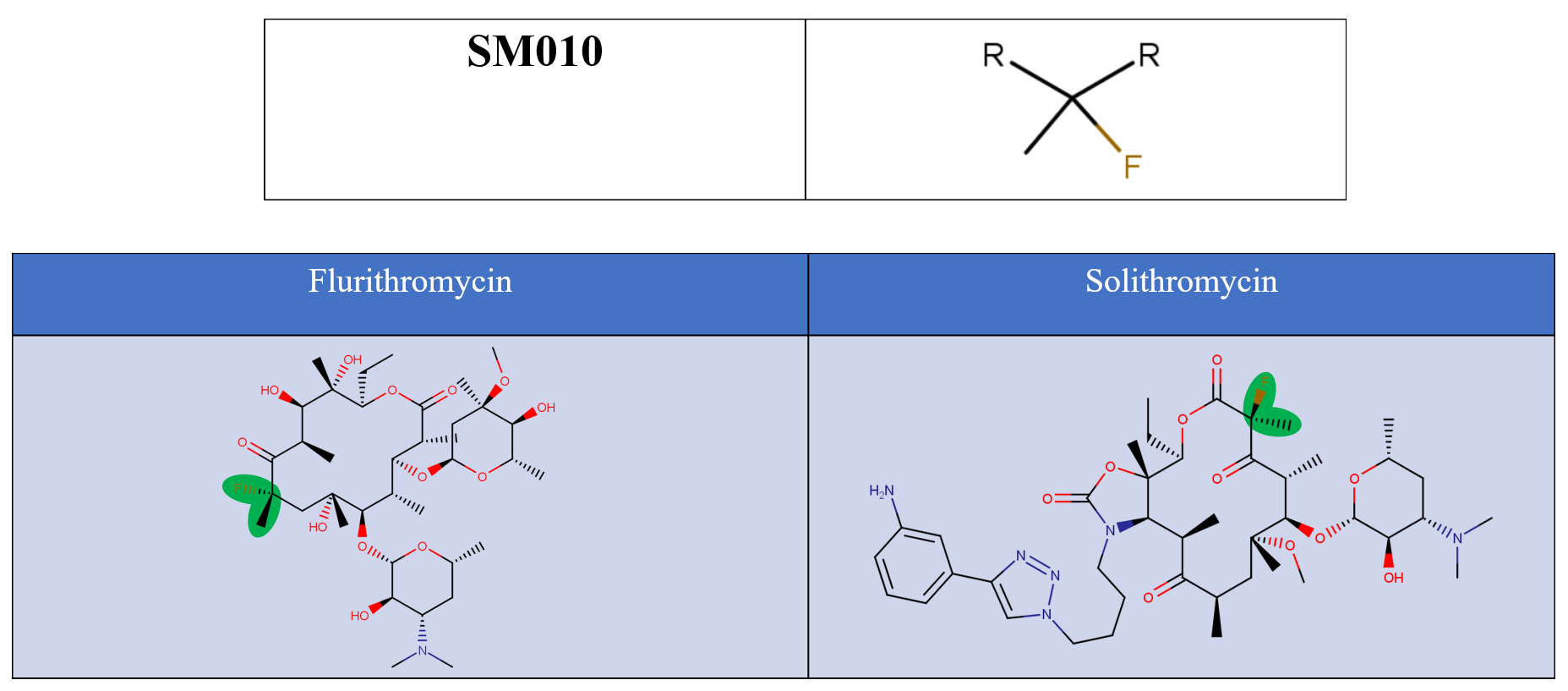


**Figure S1J.** SM010 (α-fluoro methyl) found and derived from bioactive macrolide scaffolds


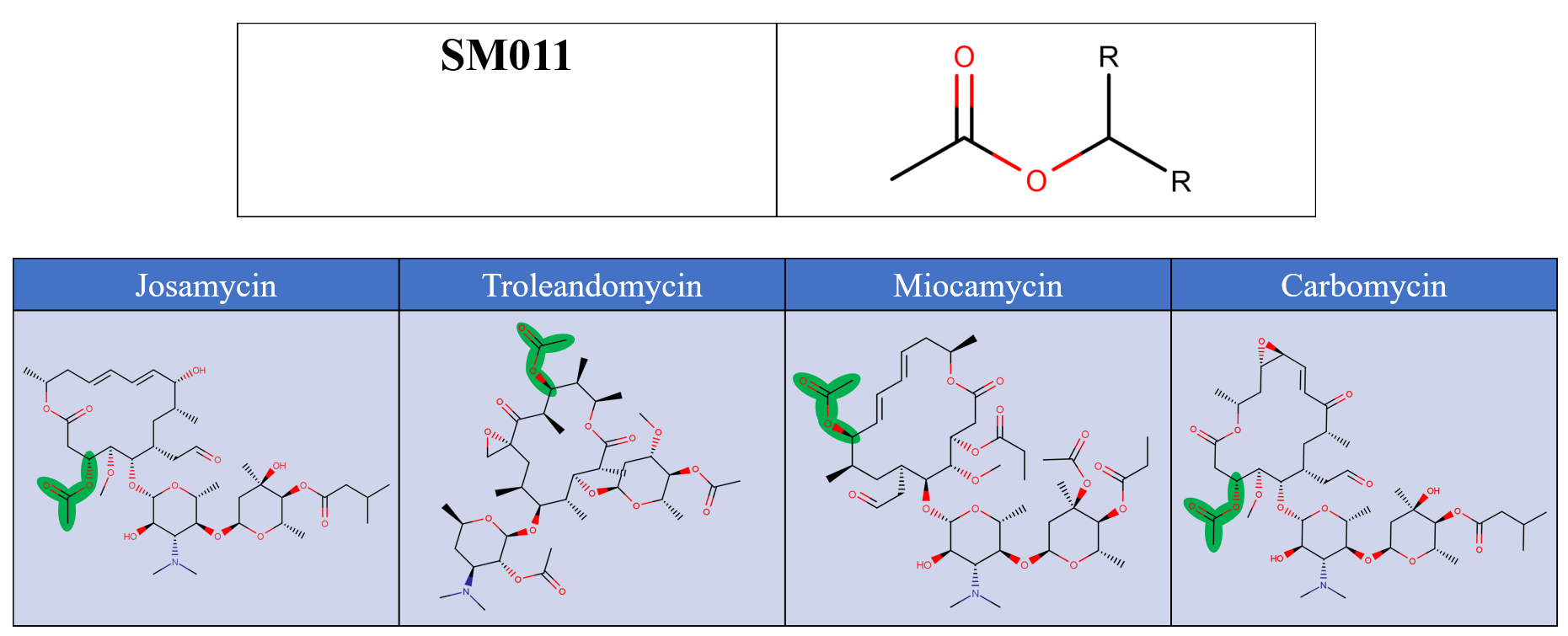


**Figure S1K.** SM011 (acetoxy) found and derived from bioactive macrolide scaffolds


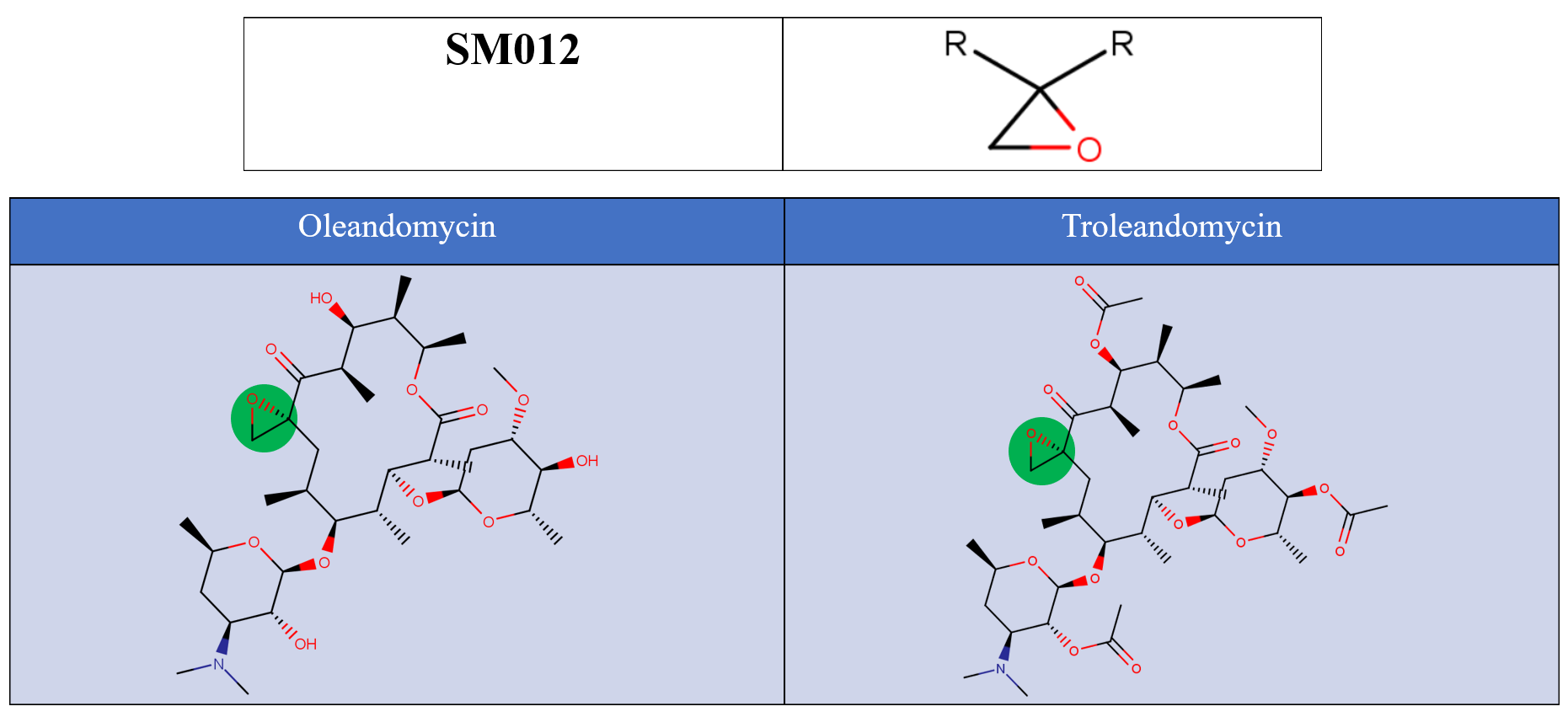


**Figure S1L.** SM012 (2,2-disubstituted epoxide) found and derived from bioactive macrolide scaffolds


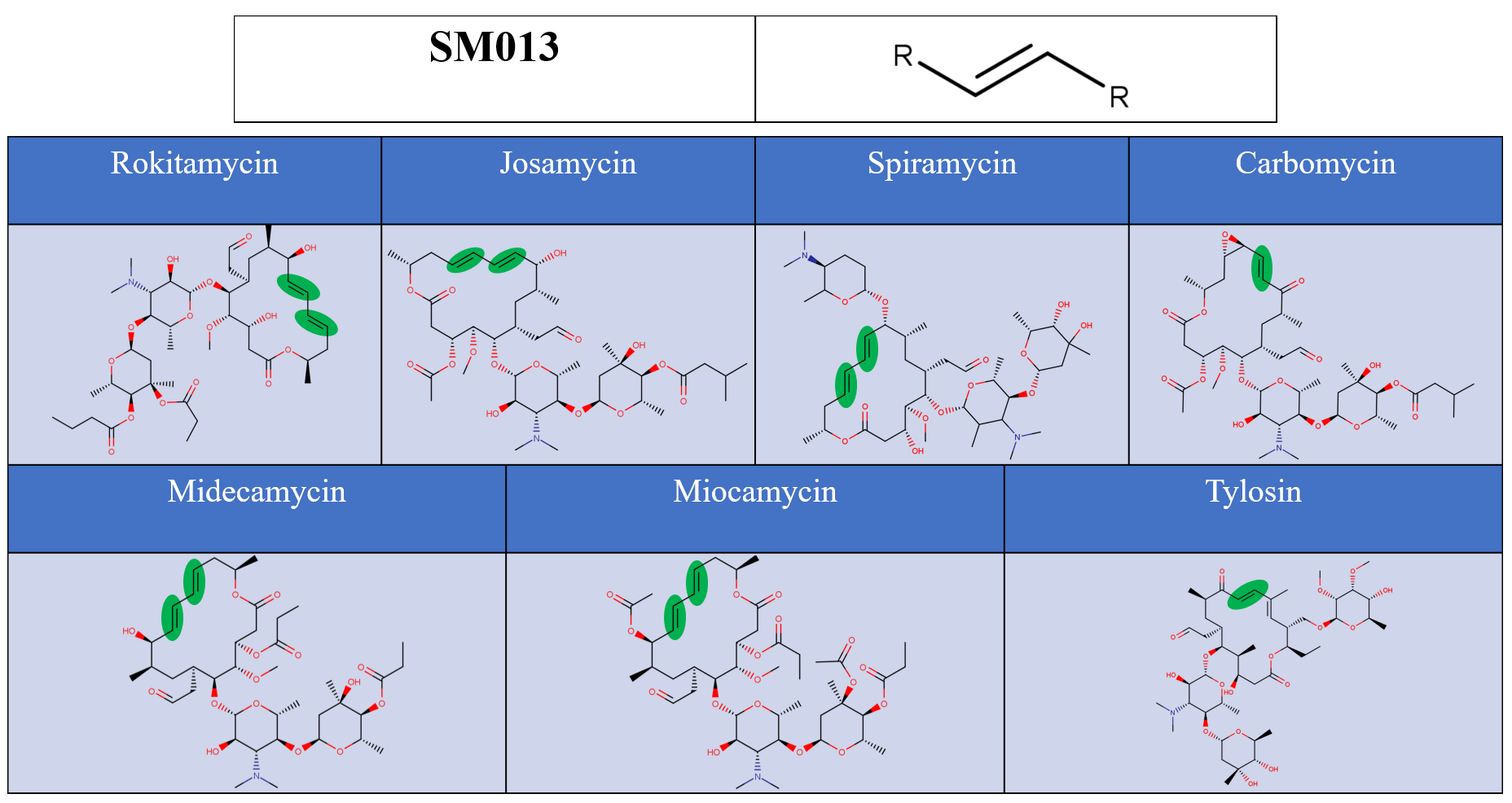


**Figure S1M.** SM013 (alkene) found and derived from bioactive macrolide scaffolds


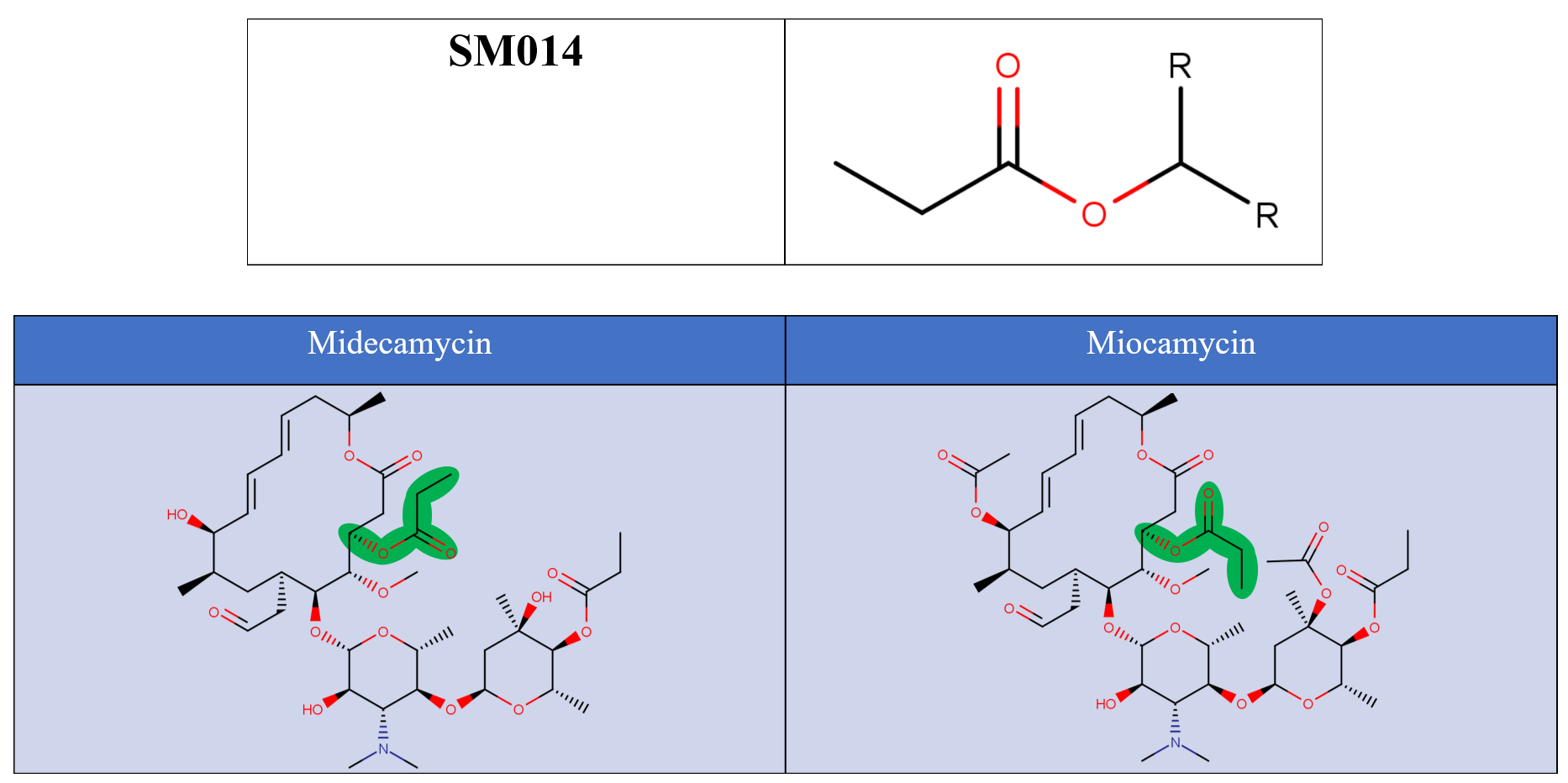


**Figure S1N.** SM014 (propionyloxy) found and derived from bioactive macrolide scaffolds


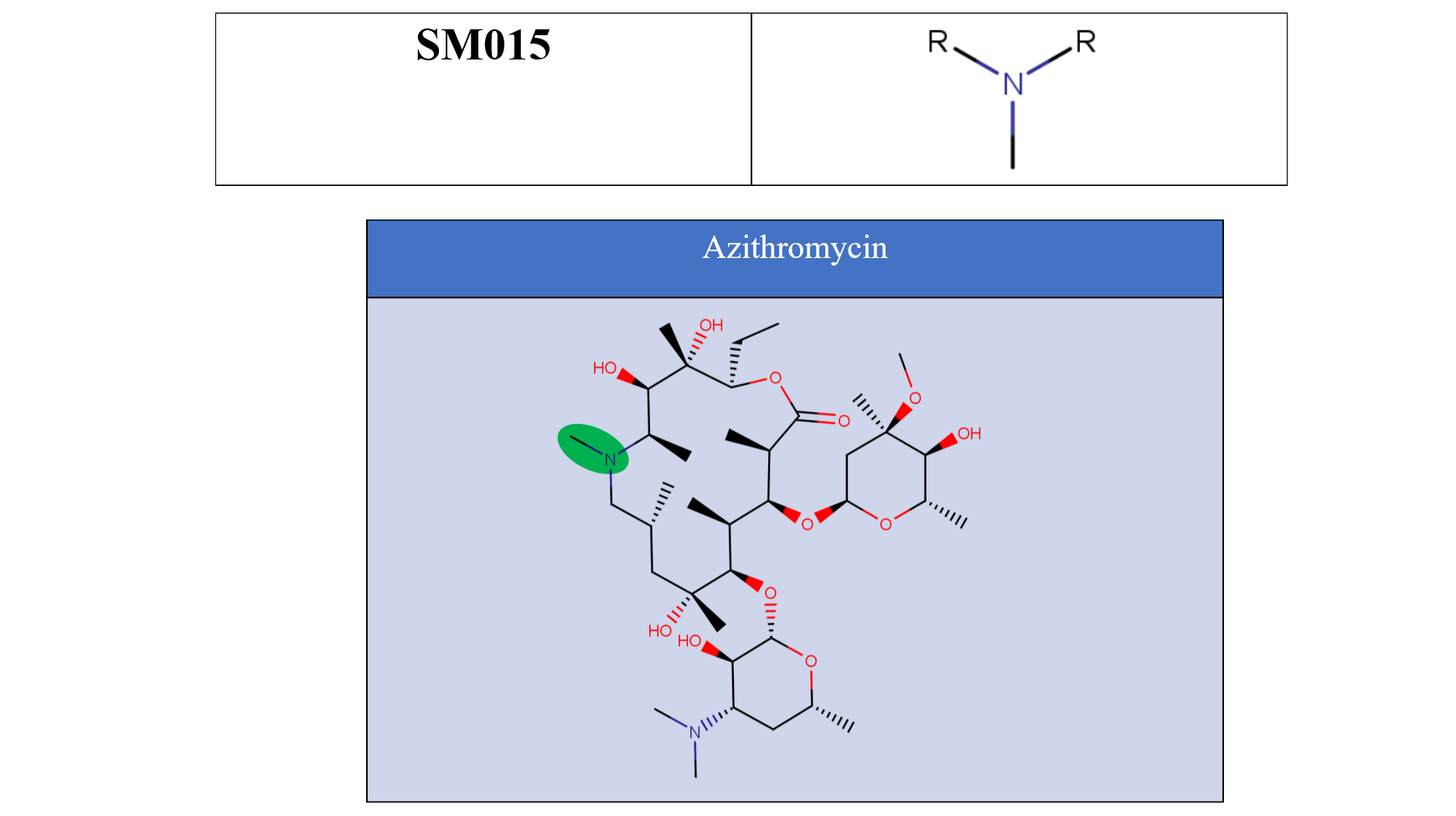


**Figure S1O.** SM015 (N-methyl amino) found and derived from bioactive macrolide scaffolds


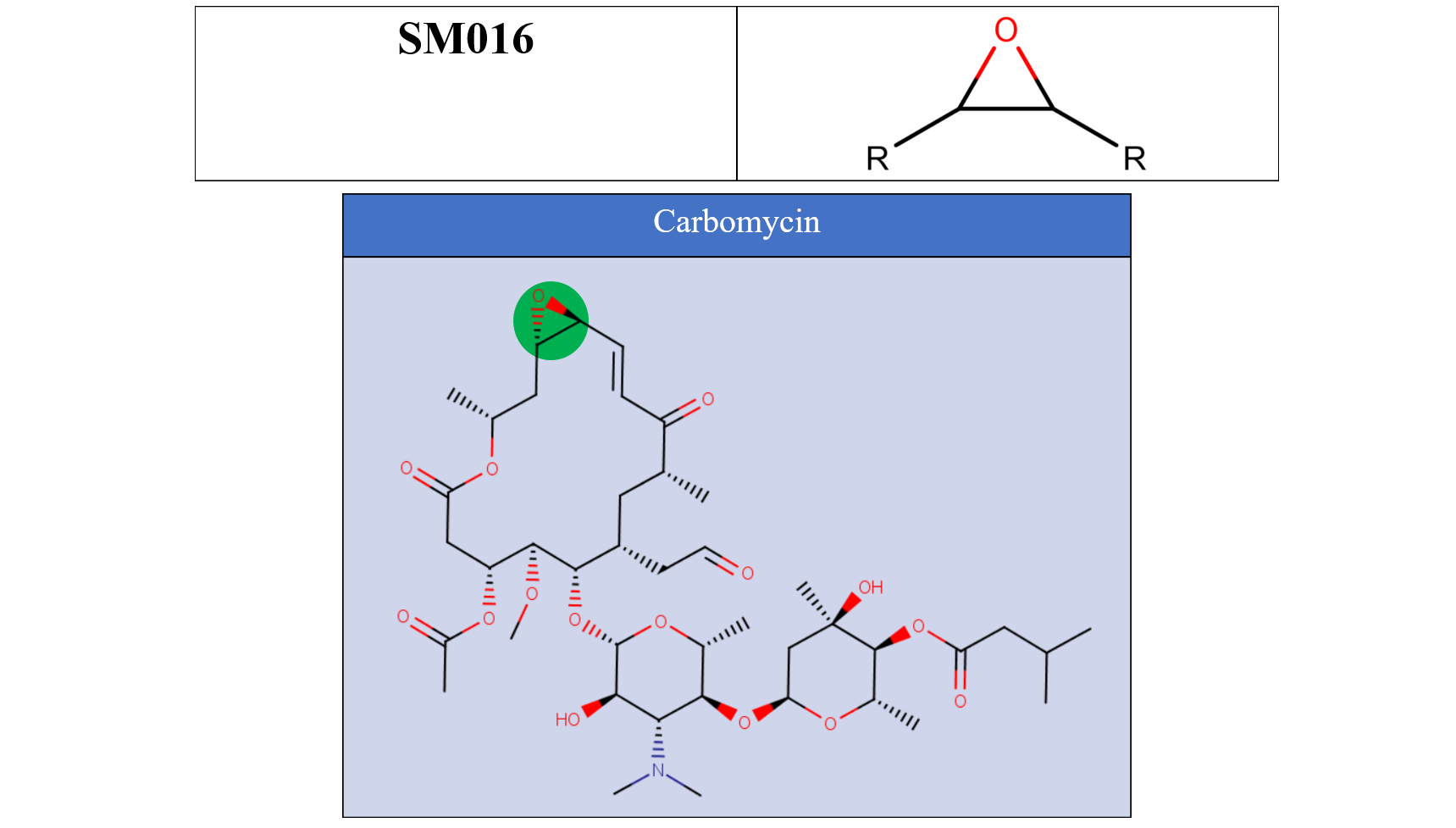


**Figure S1P.** SM016 (2,3-disubstituted epoxide) found and derived from bioactive macrolide scaffolds


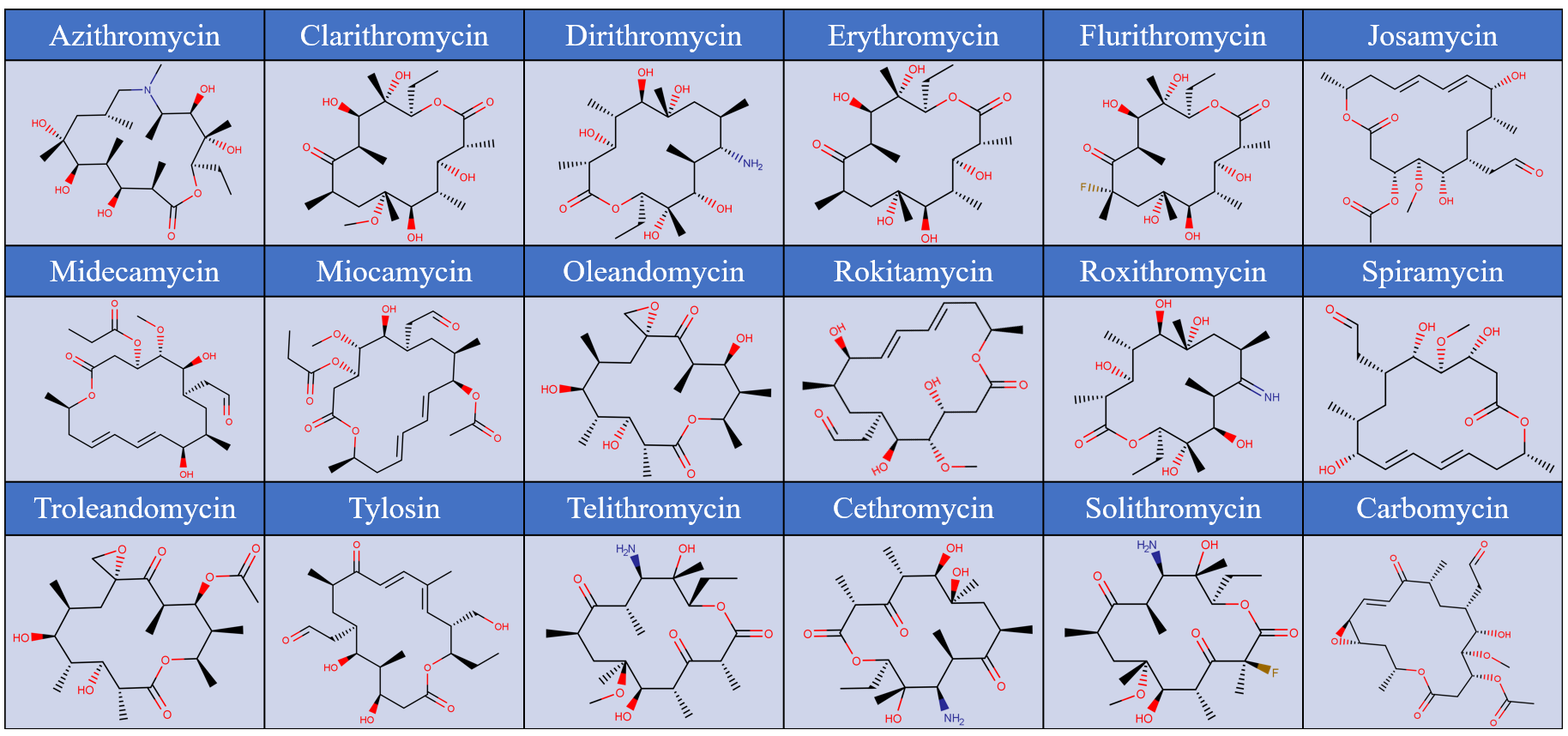


**Figure S2.** Modified structures of eighteen well-known bioactive macrolides. The original structures were simplified by removing sugar groups and replacing ester and amino chains protruding from the core cyclic structures with alcohol and amine respectively. The original structures of these macrolides are shown in **Figure 3** of the paper.


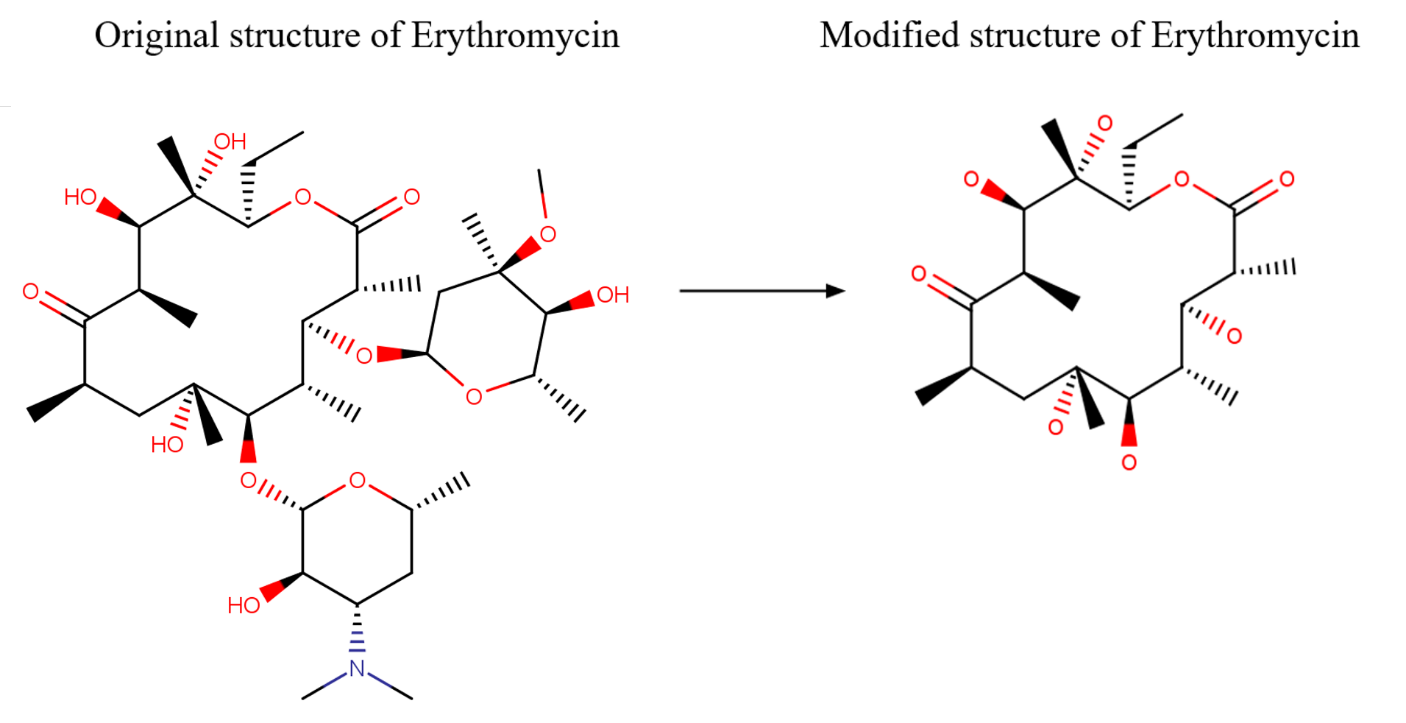


**Figure S3.** Structural simplification of Erythromycin for a comparative study with enumerated virtual macrolide scaffolds from V1M. Bulky, substituted ester and amino functional groups protruding from the ring cyclic frameworks were replaced with alcohol and amine groups respectively, and sugar blocks were removed.


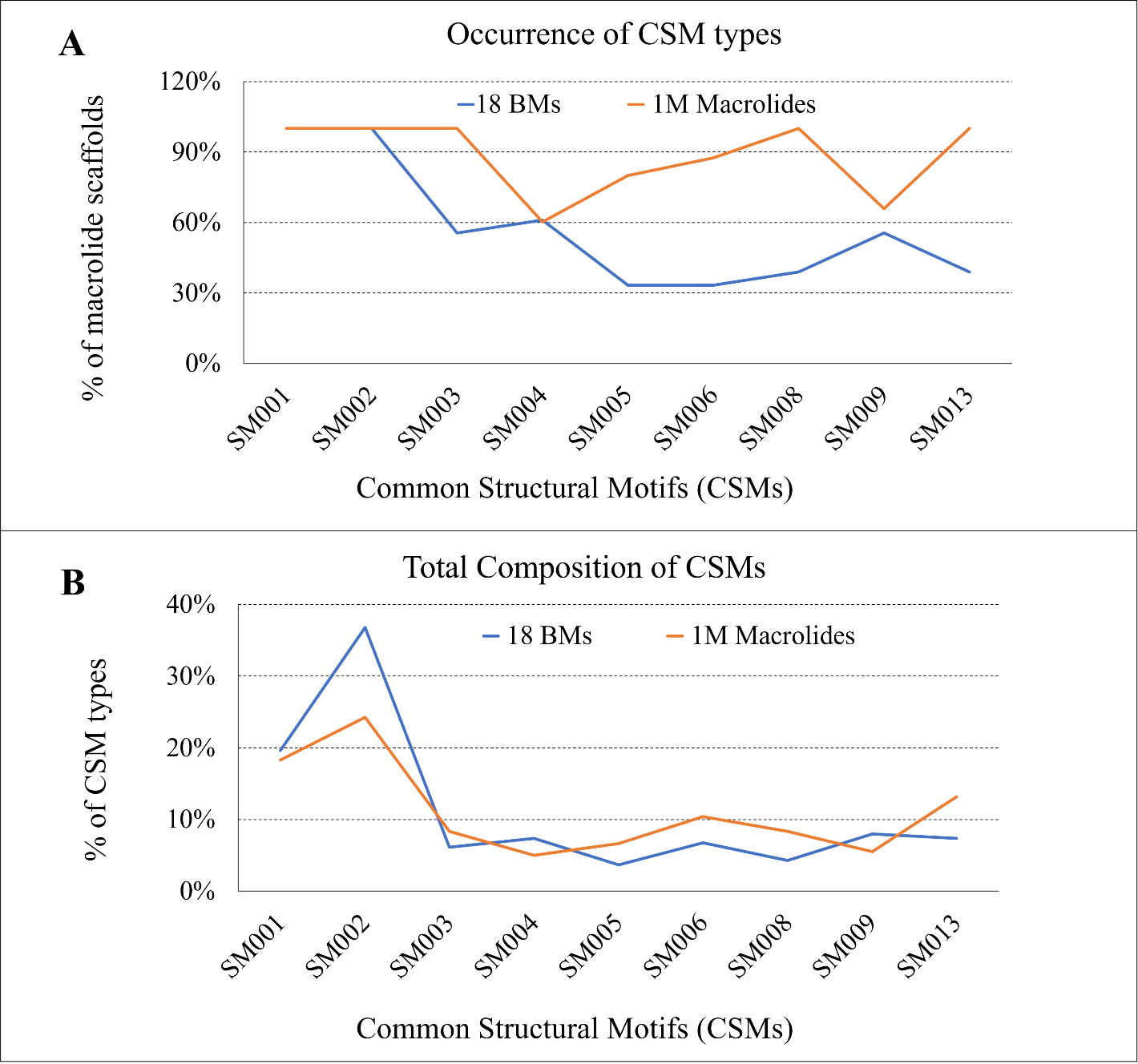


**Figure S4.** Percentage of (A) macrolide scaffolds in which associated CSM types were found, and (B) CSM type composition, in 18 BMs and V1M.


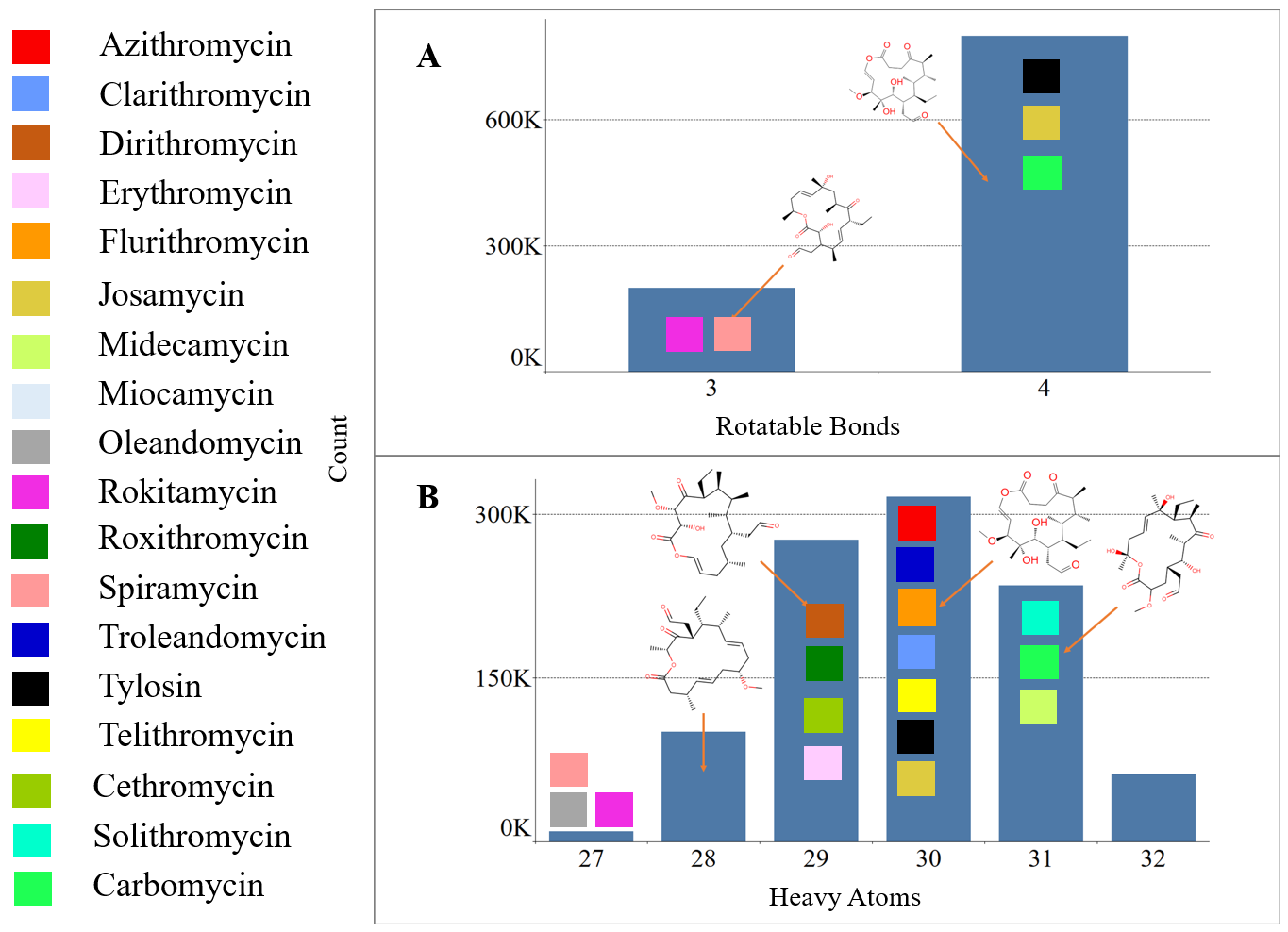


**Figure S5.** Distribution of (A) rotatable bonds, and (B) heavy atoms in V1M


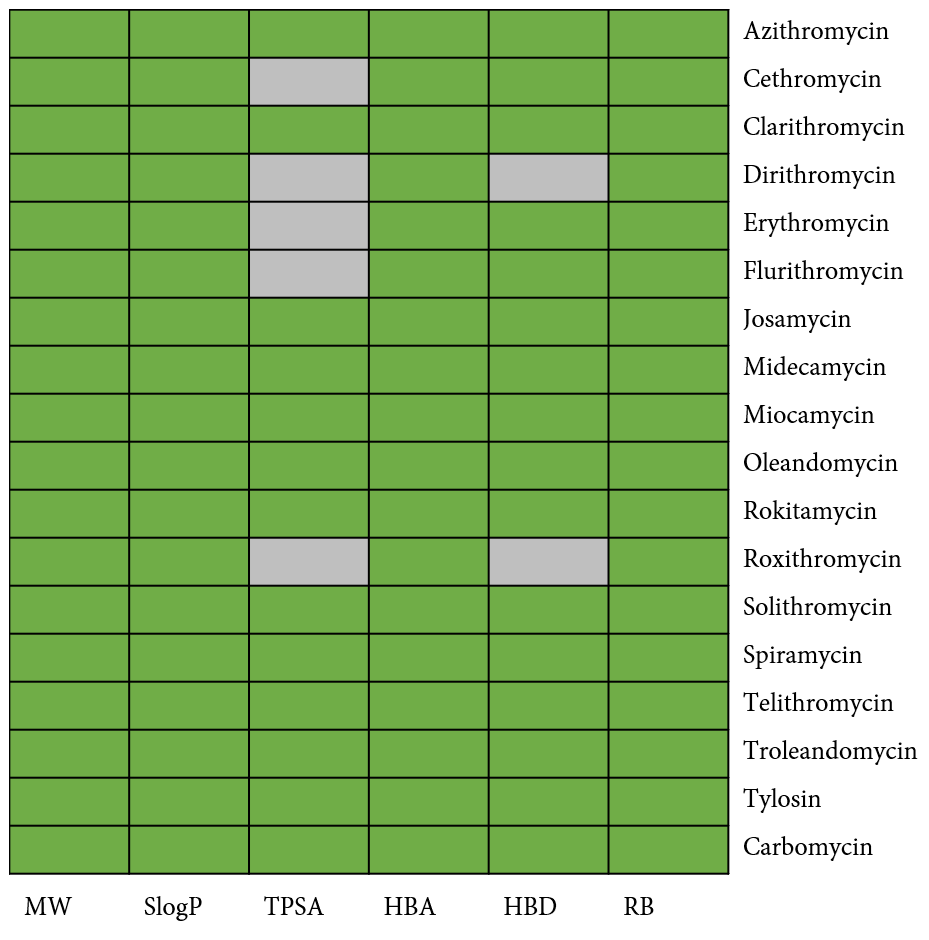


**Figure S6.** Color-coded map to demonstrate the molecular properties of eighteen bioactive macrolide scaffolds in correlation to Lipinski’s and Veber’s rules: green grids indicate compliance to the rules and grey grids indicate otherwise. Lipinski’s and Veber’s rules predict drug likeness of compounds based on the following molecular properties: MW ≤ 500, SlogP ≤ 5, TPSA ≤ 140, HBA ≤10, HBD ≤ 5 and RB ≤11.


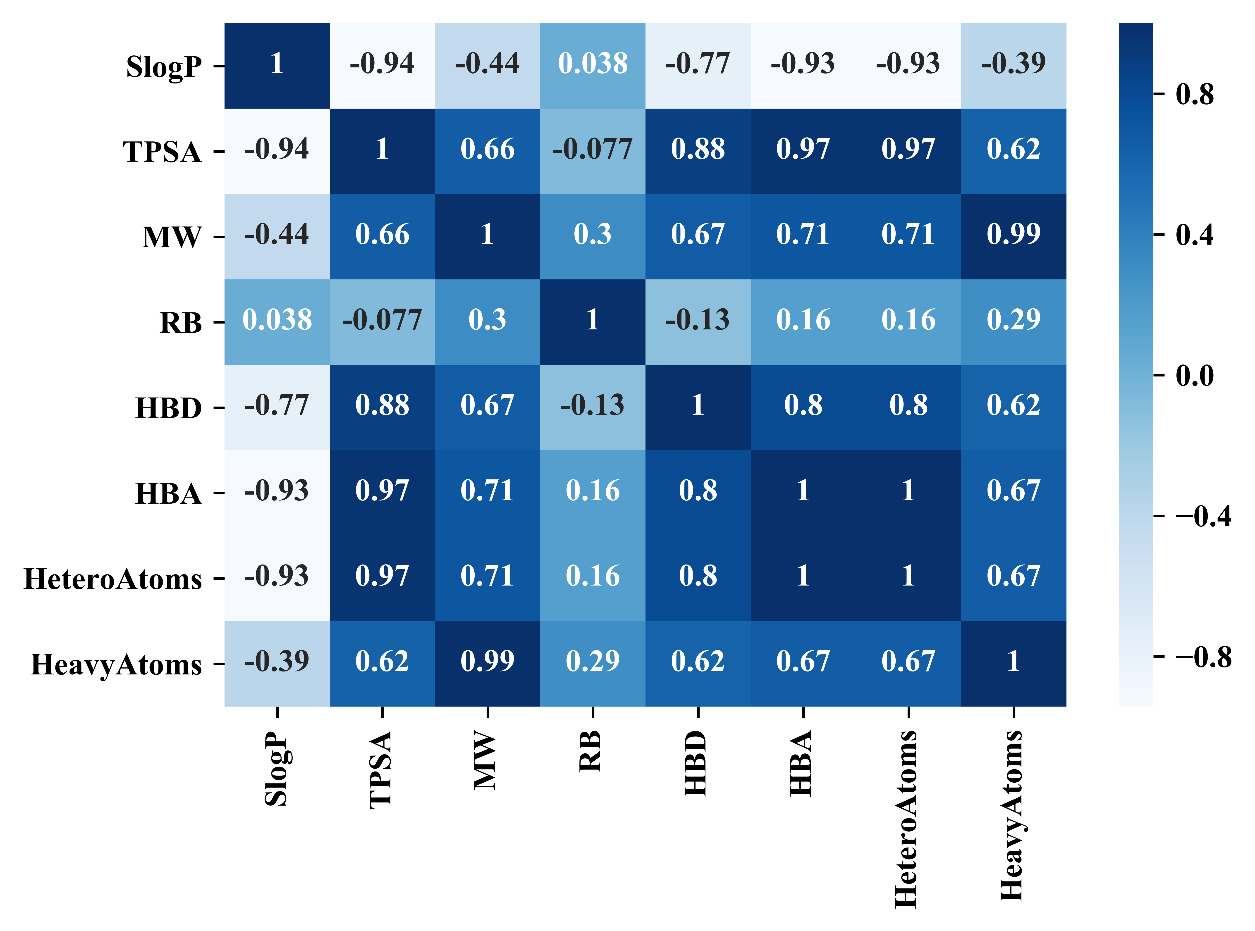


**Figure S7.** Pearson’s pair-wise correlation heatmap of all eight molecular descriptors of V1M library. Correlation values range from -1 to 1; 1 presents the strongest possible agreement, 0 no correlation and -1 the strongest possible disagreement. MW – molecular weight, SlogP – hydrophobicity, TPSA - topological polar surface area, HBA – hydrogen bond acceptors, HBD – hydrogen bond donors, RB – rotatable bonds, heteroatoms, heavy atoms


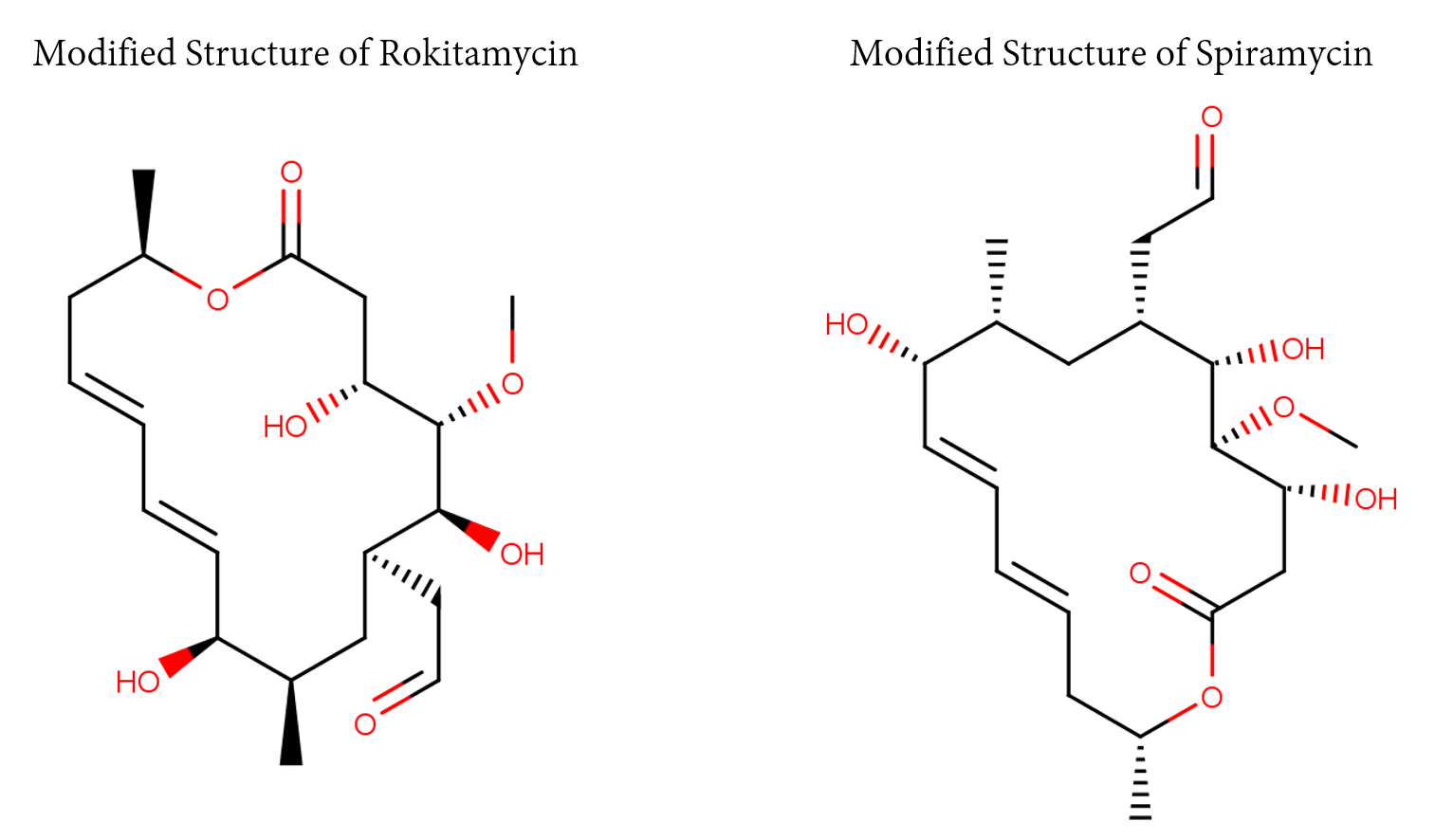


**Figure S8.** Modified structures of Rokitamycin and Spiramycin. The computed Tanimoto score between these two structures is 1, based on MACCS fingerprint method.
